# Morphological evolution of upconversion nanoparticles and their biomedical signal generation

**DOI:** 10.1038/s41598-018-35513-1

**Published:** 2018-11-20

**Authors:** Rafia Rafique, Seung Hoon Baek, Chan Yeong Park, Sung-Jin Chang, Anam Rana Gul, Siyoung Ha, Thang Phan Nguyen, Hyeongyeol Oh, Seungwook Ham, Muhammad Arshad, Hohjai Lee, Tae Jung Park

**Affiliations:** 1 Department of Chemistry, Institute of Interdisciplinary Convergence Research, Research Institute of Halal Industrialization Technology, Chung-Ang University, 84 Heukseok–ro, Dongjak–gu, Seoul 06974 Republic of Korea; 2 Department of Chemistry, School of Physics and Chemistry, Gwangju Institute of Science and Technology (GIST), 123 Cheomdan-gwagiro (Oryong-dong), Buk-gu, Gwangju 61005 Republic of Korea; 3 Institute of Environmental Sciences and Engineering, School of Civil and Environmental Engineering, National University of Sciences and Technology, Sector H-12, Islamabad, 44000 Pakistan

## Abstract

Advancements in the fabrication of upconversion nanoparticles (UCNPs) for synthetic control can enable a broad range of applications in biomedical systems. Herein, we experimentally verified the role of the hydrothermal reaction (HR) time in the synthesis of NaYF_4_:20%Yb^3+^/3%Er^3+^ UCNPs on their morphological evolution and phase transformation at different temperatures. Characterizations of the as-prepared UCNPs were conducted using X-ray diffraction (XRD), electron microscopy and spectroscopy, and thermogravimetric and upconversion (UC) luminescence analysis. We demonstrated that determining the optimal HR time, also referred to here as the threshold time, can produce particles with good homogeneity, hexagonal phase, and UC luminescence efficiency. Subsequently, the polymer coated UCNPs maintained their original particle size distribution and luminescence properties, and showed improved dispersibility in a variety of solvents, cellular nontoxicity, *in vitro* bioimaging, and biocompatibility as compared to the bare UCNP. Besides this, polyacrylic acid conjugated UCNPs (UCNP@PAA) also revealed the strong anticancer effect by conjugating with doxorubicin (DOX) as compared to the free DOX. Based on these findings, we suggest that these particles will be useful in drug-delivery systems and as *in vivo* bioimaging agents synchronously.

## Introduction

Upconversion nanoparticles (UCNPs) containing rare-earth elements have the capability to convert low-energy near-infrared (NIR) photons into higher-energy ultraviolet or visible photons^1^. The exceptional optical features of UCNPs, which are based on the NIR excitation, with deep tissue penetration and minimal autofluorescence background, underpin a broad range of applications of UCNP in diagnostics and biomedical imaging systems^2–5^. However, most UCNPs syntheses have been developed using trial-and-error approaches to achieve desirable morphologies, crystalline structures, and luminescence^6–8^. The widely used thermolysis method requires an elaborate experimental setup, high temperatures for the decomposition of organometallic precursors, and corrosive acids. In addition, this strategy leads to the formation of hydrophobic UCNPs, and further acid treatment is required to obtain oleate-ligand-free NPs^1,9,10^. Thus, more environmentally-friendly syntheses capable of producing UCNPs with controllable and reproducible morphologies, crystalline phases, and luminescence efficiencies are necessary^11^. For the synthesis of hydrophilic UCNPs, the hydrothermal method is a convenient and effective solution-based approach that can produce stable crystalline phases at considerably lower temperatures^12–14^. In this method, the crystallization process and morphological transformation of the particles can be influenced by experimental variables, such as the reaction time and temperature^15–17^, the use of organic additives^18–20^, and the pH value of precursor solution^21–23^. Reaction time and temperature are very important parameters in hydrothermal treatment. The hydrothermal reaction is conducted in a specialized reaction vessel known as an autoclave, in which a temperature gradient is maintained for a given time to obtain uniform crystal growth. Thus, it is very important to examine the effects of the hydrothermal reaction (HR) time and temperature on the UCNP synthesis process in order to determine the optimal conditions to produce UCNPs with uniform morphology, crystalline phase, and high luminescent efficiency.

In this work, we have investigated the synthesis of water-dispersible NaYF_4_:20%Yb^3+^/3%Er^3+^ UCNPs by a facile hydrothermal approach^24^. We experimentally investigated the effects of the HR time on the morphology and phase of the UCNP at different temperatures, while keeping the concentration of the dopants Yb^3+^ and Er^3+^ constant. We found that the HR time plays a dynamic role in tailoring the shape and phase of the UCNPs. Importantly, we verified that homogenous particles with a good hexagonal phase, and luminescence can be produced by optimizing the HR time. The optimized UCNPs were further functionalized with a polymer, polyacrylic acid (PAA) to increase their biocompatibility, stability, and bioconjugation *via* the presence of the non-coordinated carboxylic groups of PAA^25^. Finally, the potential of the UCNPs for use in practical biological applications has been demonstrated through the *in vitro* bioimaging of live cells, cytotoxicity, and dose response analysis of doxorubicin (DOX), one of the anti-cancer medicines, using UCNP@PAA and UCNP@PAA-DOX complexes.

## Results

### Morphology transformation

**Figure 1:**
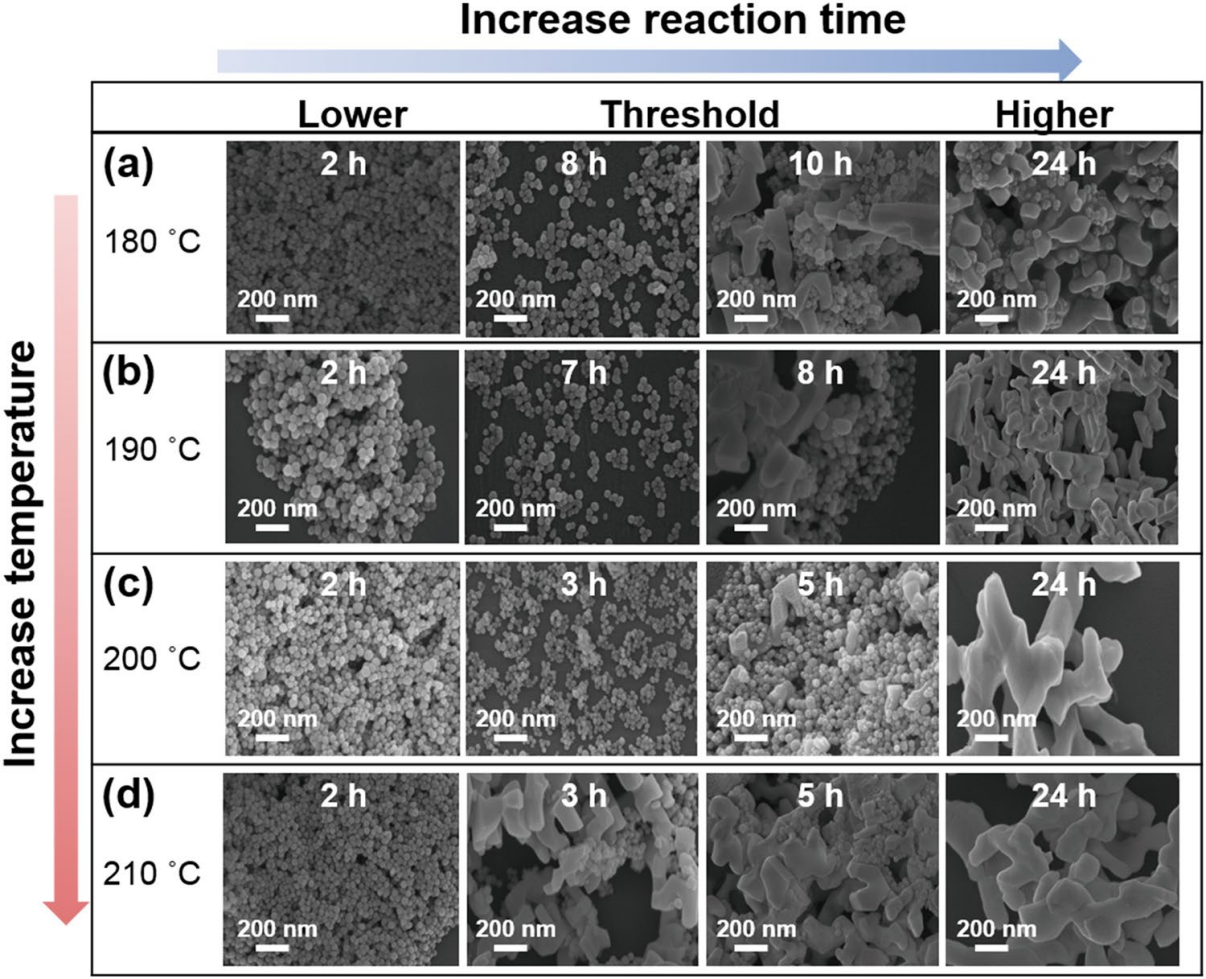
SEM images of the NaYF_4_:Yb^3+^/Er^3+^ UCNP synthesized using different HR temperatures and times. (**a**) 2, 8, 10, 24 h at 180 °C. (**b**) 2, 7, 8, 24 h at 190 °C. (**c**) 2, 3, 5, 24 h at 200 °C. (**d**) 2, 3, 5, 24 h at 210 °C.

To investigate the morphological transformation of the UCNPs during the synthesis, UCNPs synthesized at different HR times and temperatures were imaged using scanning electron microscopy (SEM, Fig. 1). Spherical UCNPs were reliably obtained at 180 °C after 2 h of HR time (Fig. 1a). This spherical shape of these UCNPs was maintained for HR times of up to 8 h. At HR times longer than 8 h, the resulting UCNPs exhibited both spherical and irregular shapes (Figs 1a and S1). After 15 h of HR, the synthesized sample consisted mainly of large and irregularly shaped UCNP (Figs 1a and S1). When the synthesis was carried out at 190 °C, the resulting UCNP exhibited a spherical shape for HR times of up to 7 h; further increasing the HR time tended to produce agglomerated particles (Figs 1b and S2). Further increases in the HR temperature decreased the HR time at which the UCNP started to show irregular shapes (Figs 1c,d and S3, S4). Increasing the HR time initially resulted in the growth of monodisperse spherical particles; however, beyond a certain HR time, large and irregular UCNPs were produced. The size distributions of the spherical UCNPs at various HR times and temperatures are presented in Figs S5–8; these results indicated that increased HR time led to an increase in the size of the UCNP (Fig. S9). Furthermore, the reproducibility of particles at different HR times and temperatures was verified by SEM and presented one more time as shown in Fig. S10.

### Structural phase transformation

**Figure 2:**
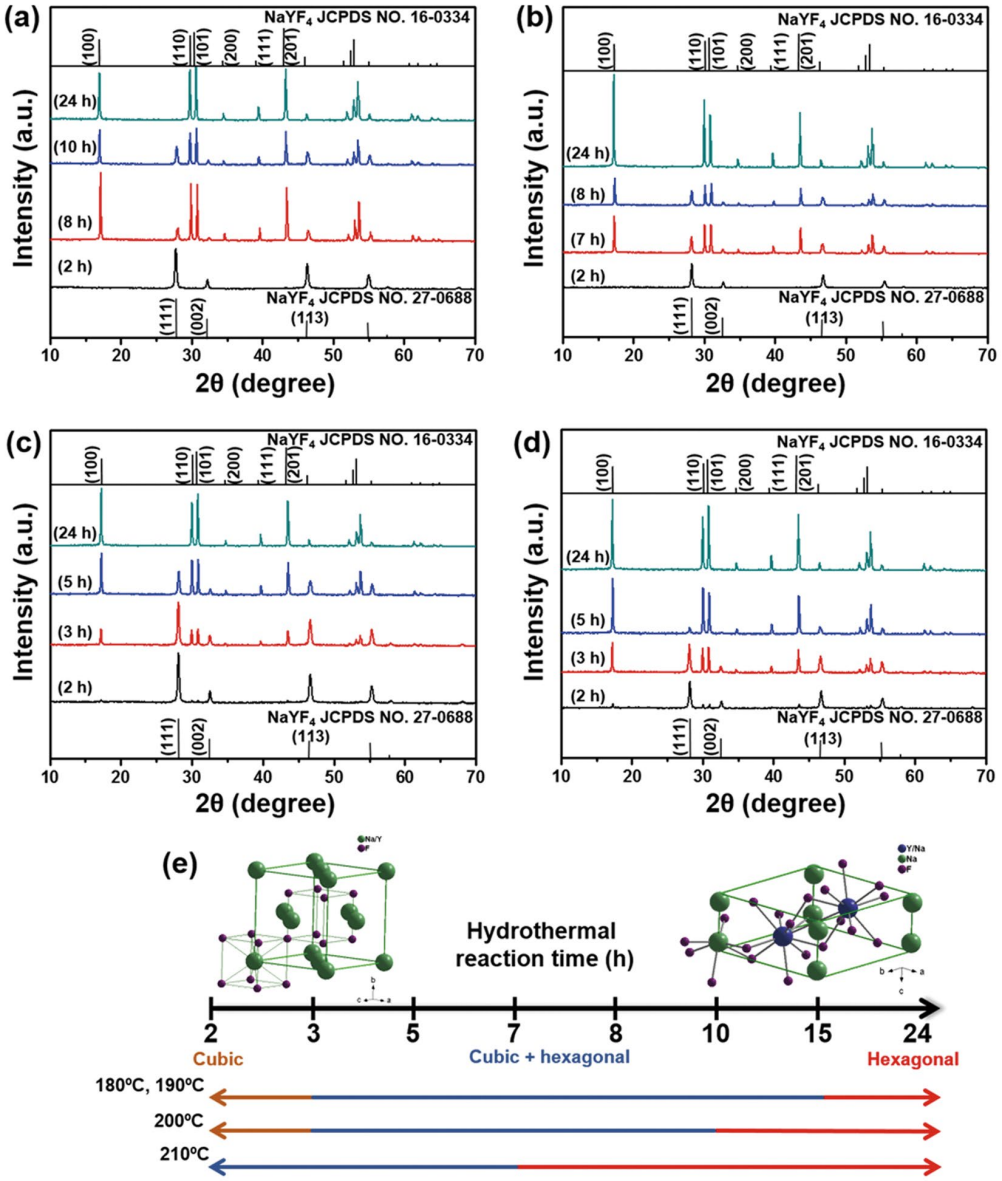
XRD patterns of the as-prepared UCNP: (**a**) 180 °C, (**b**) 190 °C, (**c**) 200 °C, (**d**) 210 °C. Particles corresponding to both α-NaYF_4_, PDF 00-027-0688 and β-NaYF_4_, PDF 00-016-0334 are observed. (**e**) Schematic illustration of the phase transformation of the UCNP with increasing HR time.

The evolution of the XRD patterns of the UCNP prepared at various HR times and temperatures is shown in Figs 2 and S11. The effect of the HR time on the evolution of the crystal phase of the UCNP is schematically illustrated in Fig. 2e. When the HR time was short (2 h), pure α-NaYF_4_ (JCPDS no. 27-0688) was obtained at 180 °C and 190 °C (Fig. 2a,b). A transition from the α-NaYF_4_ phase to a mixture of the α- and β-NaYF_4_ phases (JCPDS no. 16-0334) occurred when the HR time was increased from 3 to 15 h (Figs 2a,b and S11a,b). Pure β-NaYF_4_ was obtained after 24 h of HR at 180 °C and 190 °C (Figs 2a,b and S11a,b). However, when the temperature was increased to 200 °C or 210 °C, weak β-NaYF_4_ peaks were observed after only 2 h of HR (Fig. 2c,d). In addition, the XRD peaks of only β-NaYF_4_ phases were discernible after 15 h of HR time (Fig. S11c). The results indicated that the synthesized sample was dominated by strong β-NaYF_4_ peaks after 24 h of HR time (Fig. 2c) at a HR temperature of 200 °C. At 210 °C, the XRD peaks of α-NaYF_4_ slowly disappeared as the HR time was increased to 5 h, and those of β-NaYF_4_ became dominant after 7 h of HR (Figs 2d and S11d). A detailed analysis of the XRD peaks demonstrated that longer HR times favored the formation of pure β-NaYF_4_ from α-NaYF_4_ through a cubic-to-hexagonal crystal phase transition^26^, as schematically illustrated in Fig. 2e.

### Formation mechanism

**Figure 3:**
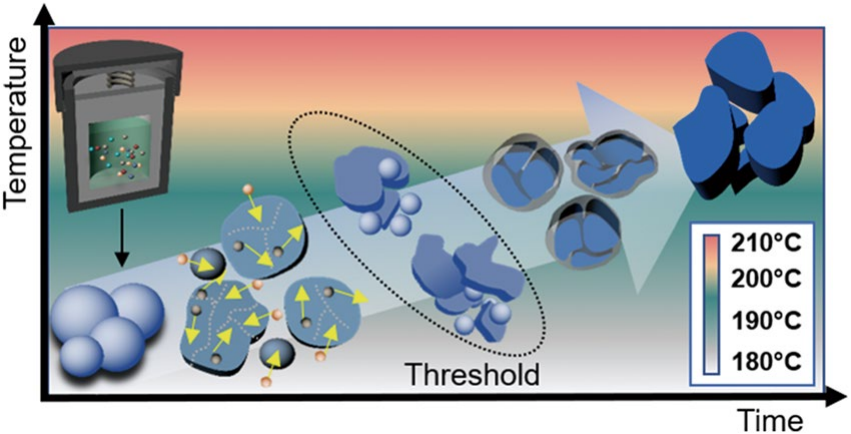
Schematic illustration of the possible growth mechanism of the NaYF_4_:Yb^3+^/Er^3+^ UCNP at different HR temperatures and times.

Based on the results shown in Fig. 1, a mechanism for the formation of the UCNP in terms of HR time and temperature is proposed (Fig. 3). During the initial stage of the synthesis, the spherical UCNPs grow larger because of possible recrystallization and Ostwald ripening processes^27^. However, after a certain HR time has elapsed, relatively large UCNPs start to form under high pressure for longer time duration^28^. The morphology transformation may occur due to the high pressure induced by the solution phase on the solid-solution phase inside the reaction vessel at HR times beyond this threshold. After the threshold HR time, the particles may be unable to withstand the high pressure and may tend to diffuse into other particles^29,30^, resulting in the formation of both spherical and irregularly shaped UCNPs due to the insufficient surface energy to form the new nanoparticles^31–33^. When the HR time is further increased, i.e., at 24 h, mainly large agglomerated particles were obtained.

The surface energy of the particles could be influenced by the phase-change inside particles which lead to the morphological changes as can be observed from high-resolution transmission electron microscopy (HR-TEM). The HR-TEM image of UCNP synthesized at 180 °C for 5 h of HR time exhibited a lattice distance of 0.31 ± 0.12 nm, which is equivalent to the *d*-spacing of the (111) plane of the α-NaYF_4_ structure (Fig. S12a). However, the particles tended to become purely hexagonal when the reaction time was increased to 24 h, as can be seen from lattice fringe of d_101_ = 0.27 ± 0.01 nm, which corresponded to the β-NaYF_4_ structure (Fig. S12b). When the reaction temperature was further increased to 190 °C, we observed *d*-spacing corresponding to (111) and (101) planes, with lattice distances of 0.32 ± 0.07 nm (α-NaYF_4_) and 0.27 ± 0.01 nm (β-NaYF_4_) at 5 h and 24 h, respectively (Fig. S12c,d). At 200 °C, the particles showed d_200_ = 0.24 ± 0.01 nm (5 h) and d_100_ = 0.48 ± 0.15 nm (24 h) lattice fringes, which were both associated with the β-NaYF_4_ structure (Fig. S12e,f). Similarly, at 210 °C, the UCNPs exhibited lattice distances of 0.48 ± 0.21 nm and 0.28 ± 0.03 nm at 5 h and 24 h, respectively (Fig. S12g,h). These results demonstrated that the β-NaYF_4_ structure became more dominant with increasing HR time, but the α-NaYF_4_ structure was present in the particles synthesized using short HR times such as 5 h, as can be seen in Figs 2 and S11^34^. Apparently, the particles were evolved from the initial α-NaYF_4_ phase to a mixed crystal phase and ultimately to the pure β-NaYF_4_ with increasing reaction time which might cause structural transformation. The HR-TEM and XRD results were comparable and showed good agreement with each other. To better understand the diffusion and distribution of the particles, the spatial relationship of the constituent elements of NaYF_4_:Yb^3+^/Er^3+^ UCNP synthesized at various temperatures for HR times of 5 h and 24 h were analyzed using elemental mapping (Figs S13–16). The elemental mapping results showed that all elements were distributed homogeneously throughout the spherical and irregularly shaped particles. In addition, the homogeneity of the elemental distribution (Y, Yb, Na, Er, and F) increased after 24 h as compared to after 5-h reaction time.

### UC luminescence properties

**Figure 4:**
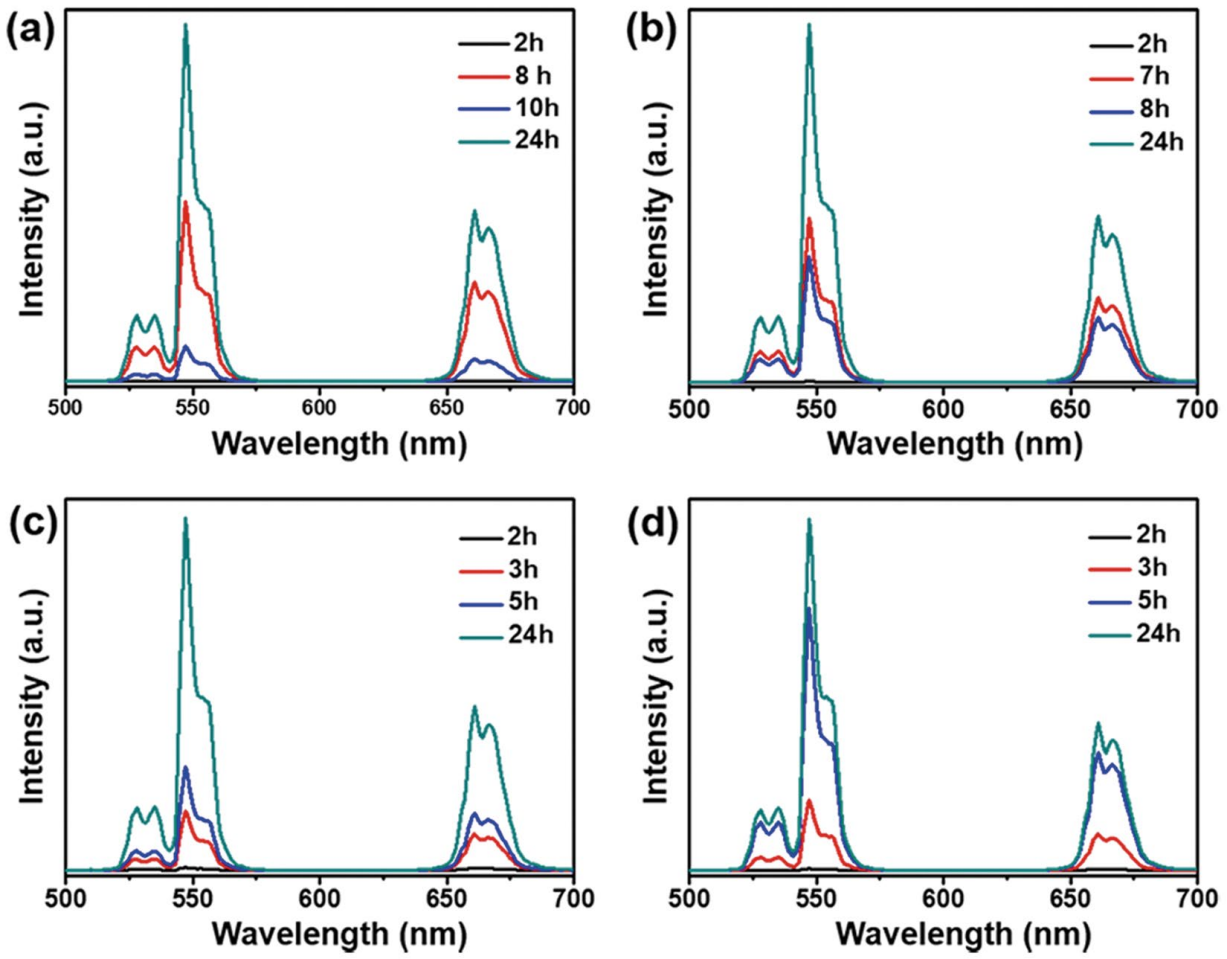
NIR to visible UC luminescence spectra of the NaYF_4_:Yb^3+^/Er^3+^ UCNP synthesized at different HR temperatures and times under 980-nm laser excitation.

The UC luminescence spectra of the UCNPs prepared by different HR times and temperatures are shown in Figs 4 and S17. The UC luminescence spectra exhibited three strong emission bands under excitation at 980 nm. These emission bands can be assigned to the ^2^H_11/2_ → ^4^I_15/2_ transition (~527 nm), the ^4^S_3/2_ → ^4^I_15/2_ transition (~540 nm), and the ^4^F_9/2_ → ^4^I_15/2_ transition (655 nm) in the Er^3+^ ions^35,36^. The enhancement of the UC luminescence with increasing HR time was mainly due to the increase in the hexagonal phase of the UCNP^37^. The UC luminescence intensity of the particles with a pure α-NaYF_4_ phase is presented in Fig. 4, black line (except at 210 °C). The luminescence intensity of particles with a mixed α and β-NaYF_4_ phase is indicated by the red and blue lines (Fig. 4a,b). However, the luminescence after 8 h and 7 h of reaction time is higher than that after 10 h and 8 h at 180 °C and 190 °C, respectively, due to the appearance of strong hexagonal peaks, as can be seen in Fig. 2a,b. Another reason may be the appearance of irregularly shaped particles, which can quench luminescence due to shape-dependent surface defects^37^. The stronger luminescence emissions after 24 h of reaction time may be due to the pure β-NaYF_4_ phase obtained^38^. These results suggest that the UC luminescence intensity depends on the crystal phase and morphology of the particles. At 200 °C and 210 °C, the UC luminescence increased with increasing hexagonal phase intensity of the UCNP as the HR time increased (Figs 4c,d and S17c,d).

To investigate different photophysical kinetics at threshold and maximum HR times and temperatures, time-resolved emission spectra of UCNPs were analyzed at the prominent green (540 nm), and red (655 nm) bands (Fig. S18a,b). The photoluminescence spectra contained both rise and decay components indicating population of the emitting ^4^S_3/2,_^4^F_9/2_ and emitted ^4^I_15/2_ states, respectively. The green emission states were populated and decayed fast as compared to red emitting states at all HR times and temperatures. This might be an outcome of differences in the green and red UC emission pathways^39^. Besides this, the efficient time raise, and decay at 24 h as compared to threshold HR times might be due to the presence of hexagonal phase with high luminescence intensity. Time-resolved photoluminescence spectra of powder UCNP samples were also recorded (Fig. S18c,d). Interestingly, the longer time raise, and decay was observed for powder samples at all HR times and temperatures as compared to solution samples. This might be due to the vibrational energy from the solvent, which can quench the luminescence emission intensity^40^.

The optimal HR time and temperature for the synthesis of UCNPs exhibiting uniform morphology (Fig. 1a), homogeneity (Fig. S5e), hexagonal phase (Fig. 2a), and strong UC luminescence (Fig. 4a) were found to be 8 h and 180 °C, respectively. The optimized UCNPs were used in the subsequent studies of cytotoxicity and bioimaging feasibility.

### Characterization of UCNP@PAA

**Figure 5:**
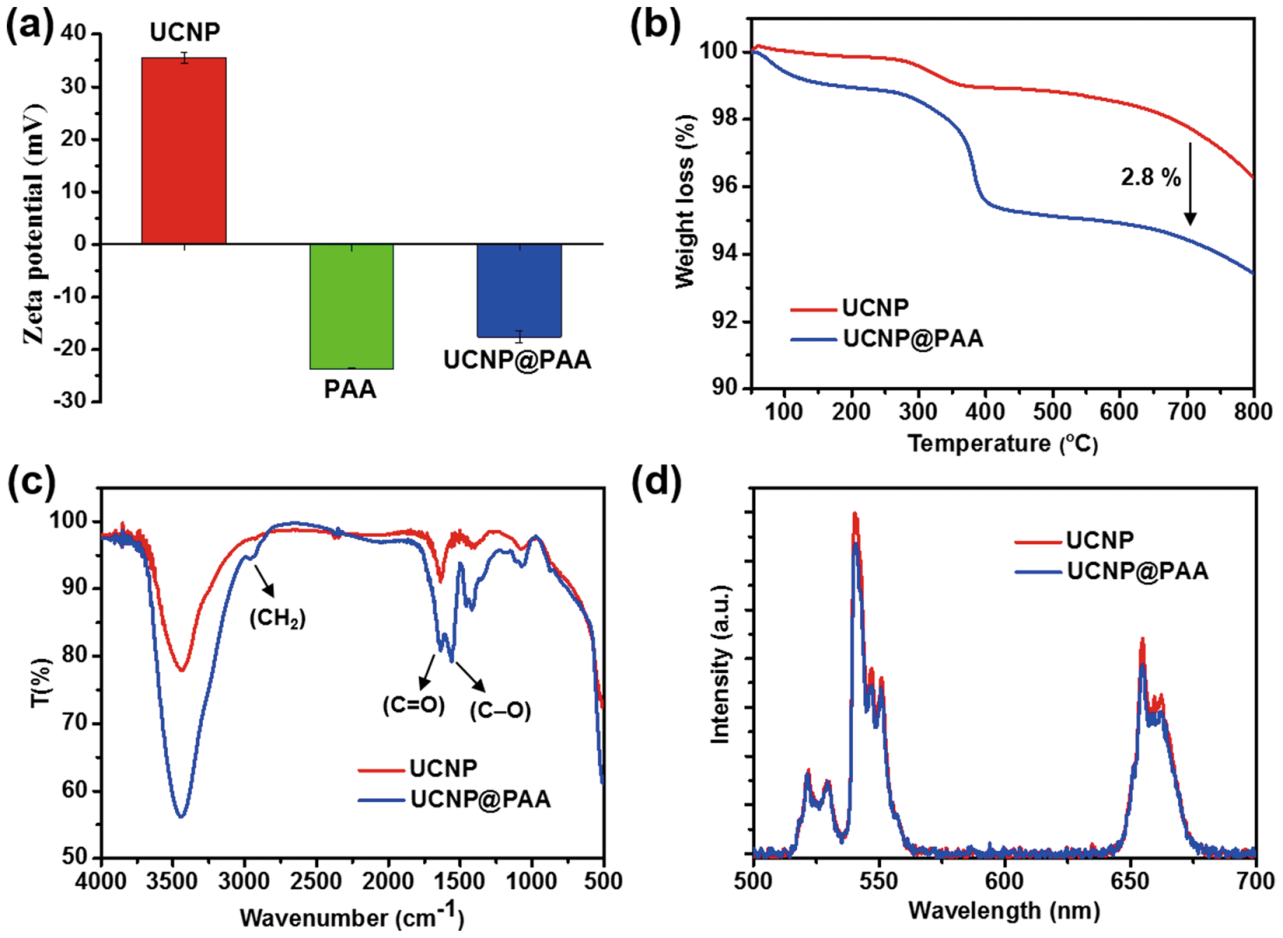
Characterization of the UCNPs after coating with PAA. (**a**) ζ potential values; insets show the UV/Vis spectra of the bared UCNP, (**b**) TGA analysis, (**c**) FTIR, and (**d**) UC luminescence spectra.

The optimized UCNPs were coated with PAA and characterized by zeta potential measurements. The zeta potential of the bare UCNPs was +36 mV, while that of the UCNPs conjugated with PAA was shifted to −18 mV (Fig. 5a). SEM images and size distribution analysis of the bare UCNP, and UCNP@PAA demonstrated the uniformity of the particles and the minimal increase in their size (Fig. S19a,b). The UV/Visible spectrum demonstrated the formation of PAA-encapsulated UCNPs (Fig. S19c). The effective attachment of PAA on the surface of UCNPs was also confirmed by the appearance of a prominent peak at around 290 nm (Fig. S19c; inset left). The inset (Fig. S19c; right) shows the UV/Visible absorption spectrum of the UCNP, which indicated the absorbance of NIR light at 980 nm. Thermogravimetric (TGA) analysis provided quantitative evidence of the successful surface modification of UCNP with PAA. The TGA data indicated that 2.8% PAA was impregnated onto the surface of the UCNPs (Fig. 5b). The presence of the PAA coating on the UCNPs was further confirmed by FTIR analysis (Fig. 5c). The FTIR spectrum of the UCNP@PAA showed absorption bands originating from PAA at 2957, 1638, and 1563 cm^−1^, which were assigned to the vibrational modes of CH_2_, C=O, and C-O, respectively^10^. Figure 5d demonstrates that the UCNPs retained their UC luminescence intensity after being coated with PAA. The long-term stability of the as-prepared UCNP and UCNP@PAA in different solvents was verified after one day and after one month (Fig. S20). The UCNP@PAA showed better dispersion in a variety of solvents after one month compared to the bare UCNP.

### *In vitro* cytotoxicity and UC luminescence imaging of cancer cells

**Figure 6:**
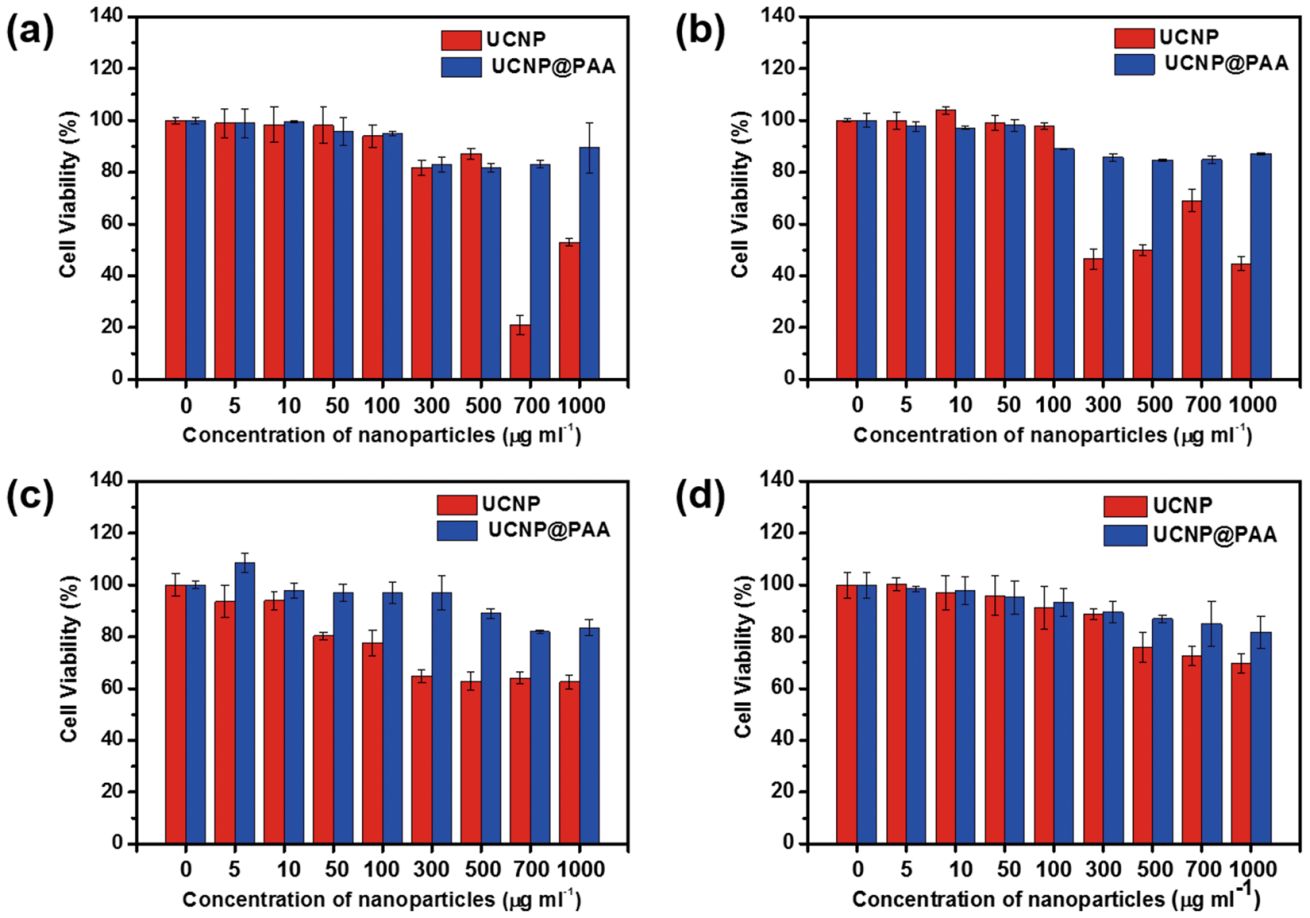
*In vitro* cytotoxicity of different concentrations of UCNP and UCNP@PAA in (**a**) HeK293, (**b**) HeLa, (**c**) A549, and (**d**) SCC7 cells after 12-h incubation time.

The as-prepared UCNPs have potential as a bioimaging agent due to their good UC luminescence efficiency, biocompatibility, and long-term stability, as shown in Fig. S20. However, the cytotoxicity of nanomaterials is an important concern in bioimaging systems. The viability of HeK293, HeLa, A549, and SCC7 cells after exposure to different concentrations of bare UCNP and UCNP@PAA was examined using a standard 3-(4,5-dimethylthiazol-2-yl)-2,5-diphenyltetrazolium bromide (MTT) assay (Fig. 6). The incubation of bare UCNP for 12 h showed negligible cytotoxicity towards HeK293, HeLa, A549, and SCC7 cells at concentration less than 700, 300, 300, and 500 μg/ml, respectively (Fig. 6a–d). The viability of the cells was reduced significantly at higher dosages. In contrast, using UCNP@PAA, the viability of the HeK293, HeLa, A549, and SCC7 cells was greater than 80% even after being exposed to 1000 μg/ml UCNP@PAA for 12 h. Thus, the UCNP@PAA were better tolerated in terms of cytotoxicity than the bare UCNP as shown by the flow cytometry analysis (Fig. S21). The UCNP@PAA were applied on HeK293, HeLa, A549, and SCC7 cells with different concentrations i.e. 300–1000 μg/ml to quantitatively analyze the anti-cancer effect. The lower necrotic and apoptotic rates were examined for the cells treated with UCNP@PAA as can be compared with the control, suggesting low cytotoxicity. The UCNP@PAA were therefore chosen for the imaging of HeLa cells due to their good stability, biocompatibility, and negligible cytotoxicity. The cells were incubated with 100 μg/ml of UCNP@PAA for 4 h, stained using DAPI, and then visualized by fluorescence microscopy under laser diode excitation at 980 nm (Fig. S22). The resulting images showed the successful uptake of the as-prepared UCNP@PAA; bright green UC luminescence spots were observed in the cytoplasm of the studied cell lines. Thus, the as-prepared UCNP@PAA has been promising as the live cell imaging agents.

### Drug loading efficiency, biocompatibility, and *in vitro* anticancer properties of UCNP@PAA-DOX

**Figure 7:**
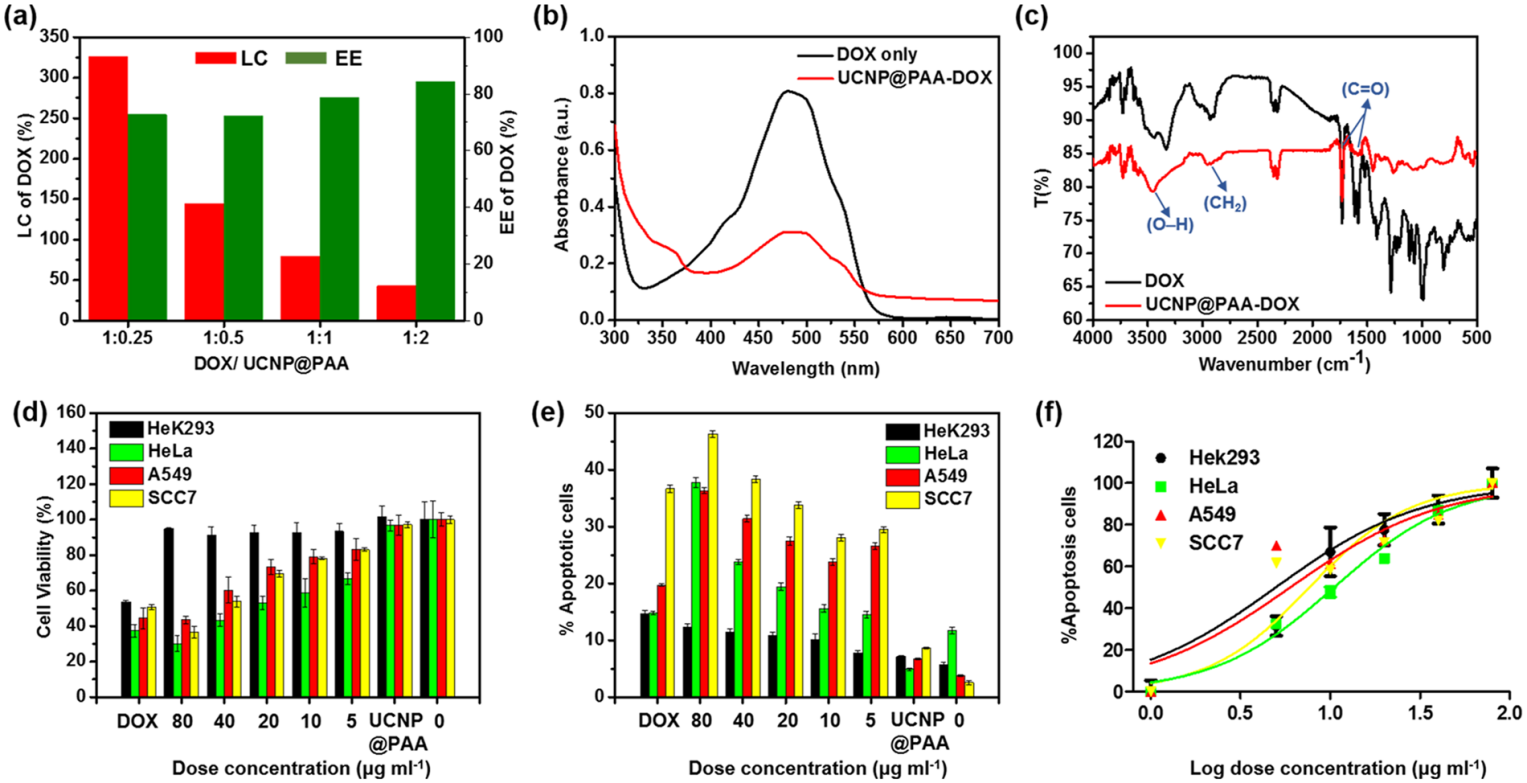
(**a**) LC and EE of DOX onto UCNP@PAA using different mass ratios, (**b**) UV/Vis, and (**c**) FTIR spectra of DOX and UCNP@PAA-DOX, (**d**) Viability of HeK293, HeLa, A549 and SCC7 cells, (**e**) % Apoptotic cell analysis using flow cytometry assay and, (**f**) IC_50_ of different cell lines in the presence of DOX, UCNP@PAA and UCNP@PAA-DOX with varied concentration from 5–80 μg/ml.

We investigated the loading capacity (LC) and encapsulation efficiency (EE) of DOX onto the UCNP@PAA with different mass ratios (DOX: UCNP@PAA-DOX = 1:0.25-2). The maximum LC and EE values of DOX on the particles surface were 325% and 72%, respectively, with a mass ratio of drug: UCNP@PAA = 1:0.25 (Fig. 7a). The results indicated that immobilization of DOX occurred during the EDC/NHS coupling, which might be originated from the interaction between carboxyl group on UCNP@PAA and amine group on DOX molecules. The UV/Visible absorption spectra displayed a red shift of PAA peak relative to UCNP@PAA (Fig. S19) and a wider DOX peak, indicating very good absorption onto the particles surface (Fig. 7b). The DOX loading is further confirmed by FTIR spectroscopy (Fig. 7c). Bands at 3450 cm^−1^ and 1732 cm^−1^ (stretching vibration of O–H and C=O) of DOX were observed along with the characteristic bands of UCNP@PAA at 2957 and 1582 cm^−1^ (stretching vibration of CH_2_ and C=O, Fig. 5c) which indicated the successful loading of drug onto the surface of UCNP@PAA.

To evaluate the potential of DOX release, 80 μg/ml of UCNP@PAA-DOX dispersed in different pH buffer solutions for various incubation times (Fig. S23). The DOX release percentage was increased in decreasing order of pH 7.4 < 6.4 < 5.3, indicating the faster release in acidic medium and with increasing incubation time. This is due to protonation of DOX amino group under acidic conditions which turn the drug more water-soluble around tumor environment and lysosome sites (pH 5.5)^41,42^. Hence, it can be inferred that amide bond breaking in weak acid environment which can accelerate the DOX release.

The therapeutic effect of UCNP@PAA-DOX was evaluated by MTT and flow cytometry assay using four cell lines including two human cancer (HeLa, A549), one mouse cancer (SCC7) and one normal human cell line (Hek293) as a negative control. The results were compared with cytotoxic effect of UCNP@PAA (80 μg/ml), different concentrations of UCNP@PAA-DOX including 80 μg/ml and DOX (60 μg/ml) only (Fig. 7d). Here, we examined the same concentration of DOX as loaded on the surface of 80 μg/ml of UCNP@PAA-DOX (1:0.25). In the absence of drug, pure UCNP@PAA had insignificant effects on cell viability of all cell lines almost equal to normal cells. Being conjugated with a natural polymer (PAA) they posed the high compatibility as a drug carrier in biological environment. In the interim, free DOX significantly reduced the viability of normal cells as compared to cancer cells. While the whole composite UCNP@PAA-DOX showed the increase of anticancer effect with increasing dose concentration on all cancer cells, and the viability of normal cells was still higher than 91%, which implied that the nanocomposite had low toxicity for normal cells (Fig. 7d). DOX shows its anticancer effect entering the cell nucleus, thus, it’s important for a drug carrier to release the drug intracellularly and protects it from degradation by enzymes and bio-environment. The anticancer effect of UCNP@PAA-DOX was further evaluated at higher level by flow cytometry. We used this assay for more precise quantitative analysis (Fig. 7e). UCNP@PAA had a least apoptotic effect on all cell lines that confirmed its highly biocompatible nature. Free DOX showed a variable dose response on different cancer cell lines. However, it also exposed the strong apoptotic behavior for normal cells, which made it weak chemotherapeutic drug for cancer treatment. DOX-loaded UCNP@PAA indicated ascending apoptotic effect with increasing dose concentration on cancer cells (Fig. 7e). The half-maximal inhibitory concentration (IC_50_) of free DOX was estimated to be about 3.07 μg/ml (Fig. S24a). The UCNP@PAA-DOX showed similar inhibition effect against all cancer cells with an estimated average IC_50_ of 7.15 μg/ml, which implied significant toxicity in reducing viability of all cancer cells than free DOX (Fig. 7f). Enhanced cytotoxic effect has specified that the UCNP@PAA-DOX nanoformulation, as a drug carrier, boosted the cellular release of DOX and bioimaging.

## Discussion

The NaYF_4_:20%Yb^3+^/3%Er^3+^ UCNPs were synthesized via a facile hydrothermal approach using different HR temperatures and times. The effect of HR time at different temperatures on the phase transformation, morphology evolution during the growth process, UC luminescence, and time-resolved emission intensity has been discussed in detail. In particular, the particles can tolerate increased HR time up to the certain limit (threshold HR time), after which they tend to agglomerate and become irregular in shape. These agglomerated particles, despite having strongest luminescence, are not applicable for drug delivery system due to very large and irregular morphology. Therefore, we selected the particles with uniform morphology, good hexagonal phase, and strong UC luminescence for further applications. When the optimized UCNP were coated with PAA, the resulting coated UCNP exhibited improved cytotoxicity in HeK293, HeLa, A549, and SCC7 cells compared to the bare UCNP, with the cell viabilities of greater than 80% even at the high dosage of 1000 μg/ml. We anticipated that the optimized morphology with high hexagonal phase intensity obtained by precisely controlling the HR time was responsible for their UC luminescence efficiency and time profiles, which makes them a promising bioimaging agent for *in vitro* cellular experiments. To be applied the synthesized particles *in vivo*, the *in vitro* biocompatibility and biodistribution should be clarified first. For this purpose, UCNP@PAA were further loaded with DOX to check the anticancer effect on different cell lines. The nanocomposite UCNP@PAA-DOX showed enhanced anticancer effect with increasing dose concentration on all cancer cells while keeping intact the normal cells as compared with free DOX. UCNP@PAA showed the high compatibility for biological environment so this whole nanocomposite can be effectively used for cancer theranostic studies as it contains DOX that is a highly used chemotherapeutic drug, and it also has luminescence property. The stronger luminescence emissions aimed at imaging for cancer diagnosis. Our findings may lead to a major step forward in the rational design of synthetic strategies for other nanomaterials for use in *in vivo* live cell experiments and drug-delivery systems.

## Method

### Materials

Yttrium nitrate hexa-hydrate (Y(NO_3_)_3_∙6H_2_O, 99.8%), ytterbium nitrate penta-hydrate (Yb(NO_3_)_3_∙5H_2_O, 99.9%), erbium nitrate penta-hydrate (Er(NO_3_)_3_∙5H_2_O, 99.9%), sodium fluoride (NaF, ≥99.0%), diethylene glycol (DEG, 99.0%), polyacrylic acid, N-(3-dimethylaminopropyl)-Nʹ-ethylcarbodimide hydrochloride (EDC), and N-hydroxy-succinimide (NHS, 98.0%) were obtained from Sigma-Aldrich (St. Louis, MO, USA). Sodium citrate (C_6_H_5_Na_3_O_7_∙2H_2_O, >99.0%) was provided by APS Biotech, Seoul. Nitric acid (HNO_3_, 60.0%) was purchased from Samchun (Seoul, Korea), ethyl alcohol (C_2_H_5_OH) was provided by Emsure (Billerca, MA, USA), and cetyltrimethylammonium bromide (CTAB, >99.0%) was obtained from Daejung (Seoul). Sodium hydroxide (NaOH) was obtained from KANTO chemical (Tokyo), and dimethylsulfoxide (DMSO, 99.9%) was provided by Alfa Aesar (Ward Hill, MA, USA). All the purchased reagents were of analytical grade. Solutions were prepared using deionized (DI) water (Direct–Q^®^ Water Purification System, Millipore, Billerica, MA, USA).

### Synthesis of NaYF_4_:Yb^3+^/Er^3+^ UCNPs

The NaYF_4_:Yb^3+^/Er^3+^ UCNPs were synthesized according to a previously reported hydrothermal method^24^. Briefly, Y(NO_3_)_3_∙6H_2_O (636 mg, 1.66 mmol), Yb(NO_3_)_3_∙5H_2_O (207 mg, 0.46 mmol), and Er(NO_3_)_3_∙5H_2_O (35 mg, 0.08 mmol) were added to a 100 ml beaker, and then sodium citrate (353 mg, 1.2 mmol) was mixed into the solution under vigorous stirring at room temperature for 30 min to form a white citrate complex. Subsequently, 3 ml of DI water, 22.5 ml of ethanol, and 150 mg of CTAB were mixed into the citrate solution under continuous stirring. Shortly thereafter, sodium fluoride (672 mg, 16.0 mmol) was added to the solution dropwise, and the solution was magnetically stirred at room temperature for another 2 h to form the crystal nuclei. After that, 1.5 ml of nitric acid was added, and the resultant solution was transferred to a 23 ml Teflon-lined autoclave and incubated at different reaction temperatures (180, 190, 200, and 210 °C) and for different reaction times (2, 3, 5, 7, 8, 10, 16, and 24 h). The resulting particles were obtained by centrifugation, washed with DI water and ethanol (1:1 v/v%), and dried at 60 °C in a dry air oven.

### Synthesis of UCNP@PAA

The synthesis of PAA-coated UCNP was carried out according to a protocol from the literature with a few modifications^10^. Typically, 50 mg of PAA (MW = 1800) was added to 9 ml DI water, and the pH was adjusted to 8 (using 0.2 M NaOH) under vigorous stirring at room temperature. Next, 1 ml of a UCNP dispersion was added dropwise, and the resultant solution was stirred for an additional 5 h. Thereafter, the water dispersion was dissolved in 10 ml of DEG, and the mixture was stirred for 1 h at 105 °C to remove the water. Finally, the mixture was transferred to the 23 ml Teflon-lined autoclave and incubated for 2 h at 160 °C. The particles were collected by centrifugation, washed with DI water and ethanol (1:1 v/v%), and dried at 60 °C in a dry air oven.

### Characterization

The size analysis and surface morphology of the synthesized particles were carried out by FE-SEM using a SIGMA instrument (Carl Zeiss, Cambridge, UK) at an accelerating voltage of 5 kV. HAADF STEM and elemental mapping were performed using a JEM-ARM2100F (JEOL, Japan) at an accelerating voltage of 200 kV. XRD patterns were recorded on a D8-Advance instrument (Bruker AXS, Berlin Germany) with a Cu Kα radiation source at λ = 1.54056 Å. The crystal phase information was measured in the range of 5° ≦ 2θ ≦ 70° at scanning rate of 0.023° min^−1^. The UV/Vis/NIR absorbance spectra were recorded on a spectrophotometer (V-670, Jasco, Tokyo, Japan). The UC luminescence spectra were analyzed using an Ocean Optics spectrophotometer (FLAME-UV-Vis, Shanghai, China) under irradiation from a 980-nm continuous wave laser diode. For time-resolved emission study, the UCNP samples (solution and powder) were excited with 7 ns pulse width from an optical parametric oscillator (OPO) system (GCR-150, 355 nm) pumped by Nd:YAG laser. The emission was detected with a photomultiplier tube for visible wavelengths (540 nm and 655 nm). The solution samples for time-resolved study were prepared by dispersing UCNPs of 60 μg/ml in DI water. The surface charge of the as-prepared particles was examined using a zeta potential analyzer (ELSZ-1000, Otsuka, Japan). FTIR spectra of the synthesized particles were recorded from 4000-750 cm^−1^ on an FTIR-6600-FV spectrometer (Jasco, Tokyo, Japan). Unless otherwise noted, characterizations of the synthesized materials were performed at room temperature. The surface modification of the UCNP with PAA was confirmed by TGA using a TGA N-1000 instrument (Scinco, Seoul, Korea) under an atmospheric environment from 40 °C to 800 °C at a heating rate of 10 °C min^−1^.

### Cell culture

HeK293, HeLa, A549 (human cancer cell lines), and SCC7 (mouse skin cancer cell lines) cells were obtained from the American Type Culture Collection (ATCC). The HeK293 and HeLa cells were cultured in Dulbecco’s Modified Eagle’s medium (DMEM), while the A549 and SCC7 cells were cultivated in Roswell Park Memorial Institute (RPMI) 1640 medium containing 1% antibiotics and 10% fetal bovine serum (FBS). The cells were cultured for the MTT-assay, flow cytometry, and cell imaging at 37 °C under a 95% air and 5.0% CO_2_-humidified atmosphere.

### *In vitro* DOX loading and release

UCNP@PAA was mixed with solution of DOX with different mass ratios (from DOX: UCNP@PAA = 1:0.25-2), followed by overnight stirring at room temperature. The DOX was loaded on the surface of UCNP@PAA *via* EDC/NHS coupling. The resulting UCNP@PAA-DOX was obtained by centrifugation and the concentration of DOX was measured in the initial drug solution and supernatant using Optizen POP spectrophotometer (Mecasys, Daejeon, Korea) based on the absorbance at 480 nm. The drug-release percentage was monitored at different incubation times and pH solutions according to a previous literature^42^. The LC and encapsulation efficiency (EE) were measured according to the following equations:

1 $${\rm{LC}}=({{\rm{W}}}_{{\rm{initial}}}-{{\rm{W}}}_{\mathrm{non}-\mathrm{encapsulated}})/{{\rm{W}}}_{{\rm{particles}}}\times 100 \% $$

2 $${\rm{EE}}=({{\rm{W}}}_{{\rm{initial}}}-{{\rm{W}}}_{\mathrm{non}-\mathrm{encapsulated}})/{{\rm{W}}}_{{\rm{initial}}}\times 100 \% $$

Where W_initial_ is the total mass of DOX; W_non-encapsulated_ is the mass of DOX after centrifugation in the supernatant; W_particles_ is the mass of UCNP@PAA added in the drug loading process.

### MTT assay

The effects of the optimized UCNP (180 °C, 8 h), UCNP@PAA, and UCNP@PAA-DOX on cell viability were investigated *in vitro* using a MTT assay. The HeK293, HeLa, A549, and SCC7 cells were seeded onto a 96-well plate with 4 × 10^3^ cells in 200 µl of culture medium per well. The plates were then incubated for 24 h at 37 °C in the presence of 5% CO_2_ to allow the cells to attach to the wells. The culture media were then replaced by a media containing different concentrations of UCNP, UCNP@PAA, and UCNP@PAA-DOX; each concentration was tested in triplicate for all cell lines. After overnight culturing at 37 °C in the presence of 5% CO_2_, MTT reagent (150 μl, 1 mg/ml) was added to each well, and then cells were incubated for a further 4 h at 37 °C. After the reaction, the color development was measured at a detection wavelength of 540 nm using a UV-Vis-IR microplate reader (BioTek Synergy H1, Winooski, VT, USA).

### Flow cytometry

To further evaluate the cellular cytotoxicity against particles, an Annexin-V fluorescein isothiocyanate (FITC)/propidium iodide (PI) procedure was applied by flow cytometry. The HeK293, HeLa, A549, and SCC7 cells were seeded onto cell culture plates (4 × 10^4^ cells per well) and then treated with different concentrations of UCNP@PAA, and UCNP@PAA-DOX. The plates were then incubated for overnight at 37 °C in the presence of 5% CO_2_. The media was subsequently removed, and cells were then resuspended with 200 μl binding buffer. After, the cells were stained with Annexin-V FITC of 5 μl and PI of 5 μl to identify apoptotic and dead cells, respectively. The cells were further incubated for 15 min and suspended with 400 μl binding buffer solution. The apoptosis induction was finally measured by analyzing 10,000 ungated cells with a BD Accuri C6 fluorescence-activated cell sorting (FACS) flow cytometer.

The HeLa cells were treated with 100 μg of UCNP@PAA for fluorescence imaging and incubated at 37 °C for 4 h in a 5.0% CO_2_ atmosphere. After incubation, the cells were washed three times with phosphate-buffered saline (PBS) to remove unbound cells and particles. Fluorescence imaging of the cells was performed using a microscope (JuLI Stage, NanoEntek, Seoul, Korea) under 980-nm laser diode excitation.

## Electronic supplementary material

Supplementary Information

## References

[1] Chen G, Qiu H, Prasad PN, Chen X (2014). Upconversion nanoparticles: Design, nanochemistry, and applications in theranostics. Chem. Rev..

[2] Xu J (2017). Near-infrared-triggered photodynamic therapy with multitasking upconversion nanoparticles in combination with checkpoint blockade for immunotherapy of colorectal cancer. ACS Nano.

[3] Kwon OS (2016). Dual-color emissive upconversion nanocapsules for differential cancer bioimaging. In Vivo. ACS Nano.

[4] Zhou B, Shi B, Jin D, Liu X (2015). Controlling upconversion nanocrystals for emerging applications. Nat. Nano.

[5] Liu B, Li C, Yang P, Hou Z, Lin J (2017). 808-nm-light-excited lanthanide-doped nanoparticles: Rational design, luminescence control and theranostic applications. Adv. Mater..

[6] Liu Y (2017). Amplified stimulated emission in upconversion nanoparticles for super-resolution nanoscopy. Nature.

[7] Yang D, Ma Pa, Hou Z, Cheng Z, Li C, Lin J (2015). Current advances in lanthanide ion (Ln^3+^)-based upconversion nanomaterials for drug delivery. Chem. Soc. Rev..

[8] Bagheri A, Arandiyan H, Boyer C, Lim M (2016). Lanthanide-doped upconversion nanoparticles: Emerging intelligent light-activated drug delivery systems. Adv. Sci..

[9] Bogdan N, Vetrone F, Ozin GA, Capobianco JA (2011). Synthesis of ligand-free colloidally stable water dispersible brightly luminescent lanthanide-doped upconverting nanoparticles. Nano Lett..

[10] Kong W (2017). A general strategy for ligand exchange on upconversion nanoparticles. Inorg. Chem..

[11] Wang F (2010). Simultaneous phase and size control of upconversion nanocrystals through lanthanide doping. Nature.

[12] Nie L (2017). Selective synthesis of LaF_3_ and NaLaF_4_ nanocrystals via lanthanide ion doping. J. Mater. Chem. C.

[13] Yang D (2013). Hollow structured upconversion luminescent NaYF_4_:Yb^3+^, Er^3+^ nanospheres for cell imaging and targeted anti-cancer drug delivery. Biomater..

[14] Wang C (2017). Multicolor tunable luminescence based on Tb^3+^/Eu^3+^ doping through a facile hydrothermal route. ACS Appl. Mater. Interfaces.

[15] Luo Y (2015). Shape-controllable hydrothermal synthesis of NaTbF_4_:Eu^3+^ microcrystals with energy transfer from Tb to Eu and multicolor luminescence properties. Cryst Eng Comm.

[16] Zeng S, Ren G, Li W, Xu C, Yang Q (2010). Highly uniform Tm^3+^-doped NaYbF_4_ microtubes: Controlled synthesis and intense ultraviolet photoluminescence. J. Phys. Chem. C.

[17] Zhang F (2007). Uniform nanostructured arrays of sodium rare-earth fluorides for highly efficient multicolor upconversion luminescence. Angew. Chem. Int. Ed..

[18] Ren J, Jia G, Guo Y, Wang A, Xu S (2016). Unraveling morphology and phase control of NaLnF_4_ upconverting nanocrystals. J. Phys. Chem. C.

[19] Liu D (2016). Three-dimensional controlled growth of monodisperse sub-50 nm heterogeneous nanocrystals. Nat. Commun..

[20] Wu S, Liu Y, Chang J, Zhang S (2014). Ligand dynamic effect on phase and morphology control of hexagonal NaYF_4_. CrystEngComm.

[21] Li J (2013). Hydrothermal synthesis and upconversion luminescence properties of β-NaGdF_4_:Yb^3+^/Tm^3+^ and β-NaGdF_4_:Yb^3+^/Ho^3+^ submicron crystals with regular morphologies. J. Colloid Interface Sci..

[22] Li C, Yang J, Quan Z, Yang P, Kong D, Lin J (2007). Different microstructures of β-NaYF_4_ fabricated by hydrothermal process:  Effects of pH values and fluoride sources. Chem. Mater..

[23] Zhang Y (2013). Rapid, large-scale, morphology-controllable synthesis of YOF:Ln^3+^ (Ln = Tb, Eu, Tm, Dy, Ho, Sm) nano-/microstructures with multicolor-tunable emission properties. Inorg. Chem..

[24] Choi SY (2017). Synthesis of upconversion nanoparticles conjugated with graphene oxide quantum dots and their use against cancer cell imaging and photodynamic therapy. Biosens. Bioelectron..

[25] Liu B (2015). Poly (acrylic acid) modification of Nd^3+^-sensitized upconversion nanophosphors for highly efficient UCL imaging and pH-responsive drug delivery. Adv. Funct. Mater..

[26] Wang M, Zhu Y, Mao C (2015). Synthesis of NIR-responsive NaYF_4_:Yb,Er upconversion fluorescent nanoparticles using an optimized solvothermal method and their applications in enhanced development of latent fingerprints on various smooth substrates. Langmuir.

[27] Chen X, Peng D, Ju Q, Wang F (2015). Photon upconversion in core-shell nanoparticles. Chem. Soc. Rev..

[28] Mai H-X (2006). High-quality sodium rare-earth fluoride nanocrystals:  Controlled synthesis and optical properties. J. Am. Chem. Soc..

[29] Jose-Yacaman M (2005). Surface diffusion and coalescence of mobile metal nanoparticles. J. Phys. Chem. B.

[30] Fan HJ, Gosele U, Zacharias M (2007). Formation of nanotubes and hollow nanoparticles based on Kirkendall and diffusion processes: a review. Small.

[31] Pan Q, Yang D, Kang S, Qiu J, Dong G (2016). Regulating mid-infrared to visible fluorescence in monodispersed Er^3+^-doped La_2_O_2_S(La_2_O_2_SO_4_) nanocrystals by phase modulation. Sci. Rep..

[32] Andrievski R (2014). Review of thermal stability of nanomaterials. J. Mater. Sci..

[33] Xu L, Liang H-W, Yang Y, Yu S-H (2018). Stability and reactivity: Positive and negative aspects for nanoparticle processing. Chem. Rev..

[34] Yin B, Zhou W, Long Q, Li C, Zhang Y, Yao S (2014). Salt-assisted rapid transformation of NaYF_4_:Yb^3+^,Er^3+^ nanocrystals from cubic to hexagonal. CrystEngComm.

[35] Dong H, Sun L-D, Feng W, Gu Y, Li F, Yan C-H (2017). Versatile spectral and lifetime multiplexing nanoplatform with excitation orthogonalized upconversion luminescence. ACS Nano.

[36] Shao B (2014). A novel synthetic route towards monodisperse β-NaYF_4_:Ln^3+^ micro/nanocrystals from layered rare-earth hydroxides at ultra low temperature. Chem. Commun..

[37] Lin M (2014). Synthesis of upconversion NaYF_4_:Yb^3+^,Er^3+^ particles with enhanced luminescent intensity through control of morphology and phase. J. Phys. Chem. C.

[38] Wang F, Wang J, Liu X (2010). Direct evidence of a surface quenching effect on size-dependent luminescence of upconversion nanoparticles. Angew. Chem..

[39] Jung T (2015). The preferred upconversion pathway for the red emission of lanthanide-doped upconverting nanoparticles, NaYF_4_:Yb^3+^, Er^3+^. Phys. Chem. Chem. Phys..

[40] Rabouw, F. T., Prins, P. T., Villanueva-Delgado, P., Castelijns, M., Geitenbeek, R. G., Meijerink, A. Quenching pathways in NaYF_4_: Er^3+^, Yb^3+^ upconversion nanocrystals. *ACS Nano*, (2018). 10.1021/acsnano.8b01545 PMC5968434 29648802

[41] Cao J (2014). Polymeric micelles with citraconic amide as pH-sensitive bond in backbone for anticancer drug delivery. Int. J. Pharm..

[42] Lai CW, Hsiao YH, Peng YK, Chou PT (2012). Facile synthesis of highly emissive carbon dots from pyrolysis of glycerol; gram scale production of carbon dots/mSiO_2_ for cell imaging and drug release. J. Mater. Chem..

