# Supplementary material for: Morphological evolution of upconversion nanoparticles and their biomedical signal generation
Source: Sci Rep. 2018 Nov 20;8:17101. doi: 10.1038/s41598-018-35513-1 (PMC6244231; doi:10.1038/s41598-018-35513-1)
Supplement: Supplementary file 1 — Supplementary Information [file 41598_2018_35513_MOESM1_ESM.pdf]

Supplementary Information for

Morphological evolution of upconversion nanoparticles and their biomedical signal generation

Rafia Rafique[1], Seung Hoon Baek[1], Chan Yeong Park[1], Sung-Jin Chang[1], Anam Rana Gul[1], Siyoung Ha[1], Thang Phan Nguyen[1], Hyeongyeol Oh[2], Seungwook Ham[1], Muhammad Arshad[3], Hohjai Lee[2], and Tae Jung Park[1,*]

[1]Department of Chemistry, Institute of Interdisciplinary Convergence Research, Research Institute of Halal Industrialization Technology, Chung-Ang University, 84 Heukseok–ro, Dongjak–gu, Seoul 06974, Republic of Korea

[2]Department of Chemistry, School of Physics and Chemistry, Gwangju Institute of Science and Technology (GIST), 123 Cheomdan-gwagiro (Oryong-dong), Buk-gu, Gwangju 61005, Republic of Korea

[3]Institute of Environmental Sciences and Engineering, School of Civil and Environmental Engineering, National University of Sciences and Technology, Sector H-12, Islamabad, 44000, Pakistan

* Correspondence and requests for materials should be addressed to T.J.P. (email: tjpark@cau.ac.kr).

SEM images of UCNPs at different hydrothermal reaction times and temperatures

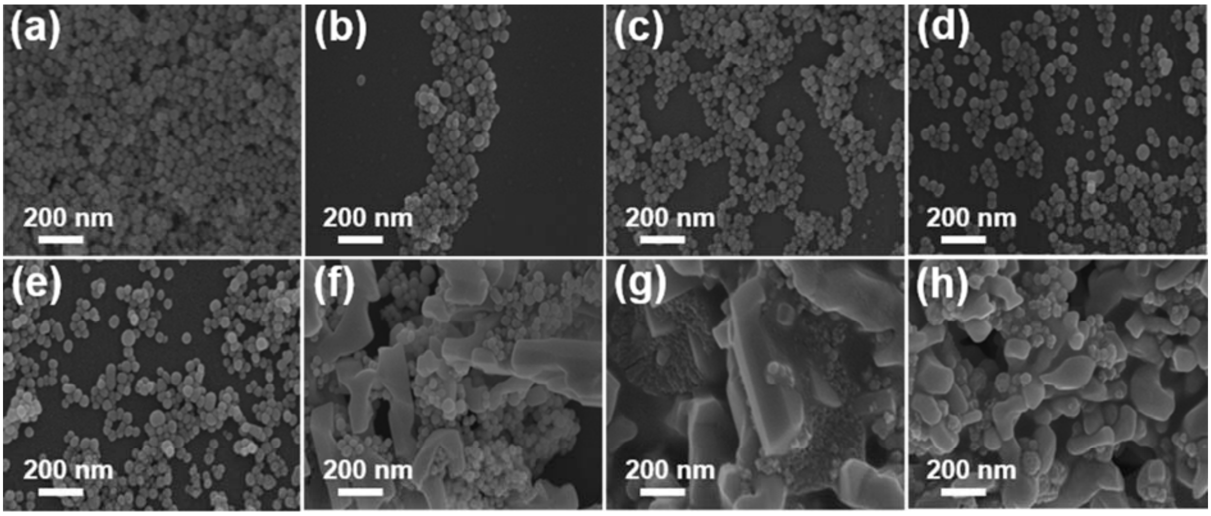

Fig. S1. SEM images of $NaYF_4:Yb^{3+}/Er^{3+}$ UCNP synthesized at 180 °C as a function of different hydrothermal reaction times, (a) 2 h, (b) 3 h, (c) 5 h, (d) 7 h, (e) 8 h, (f) 10 h, (g) 15 h, and (h) 24 h.

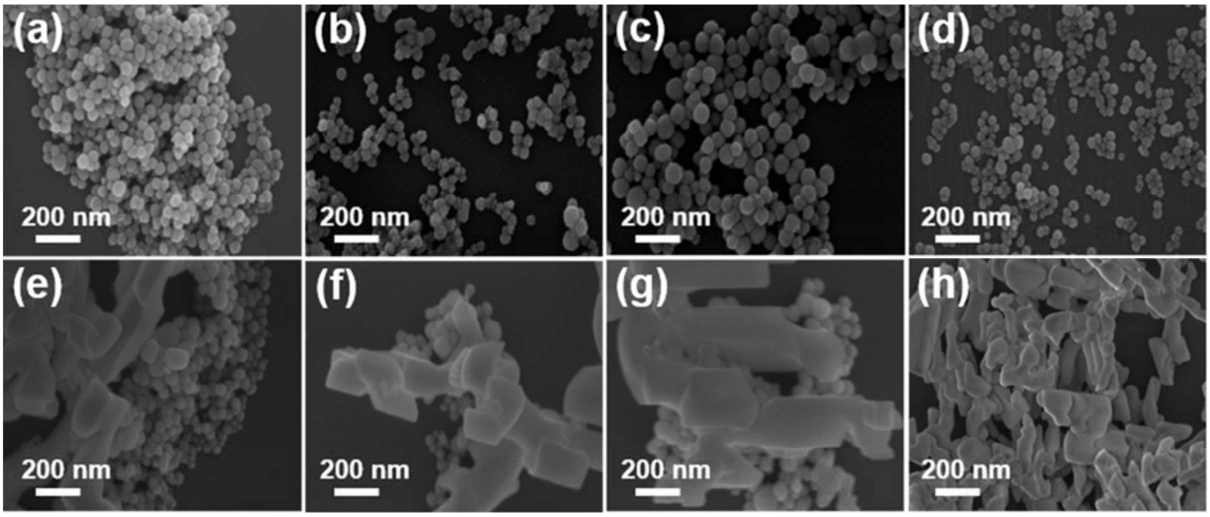

Fig. S2. SEM images of $NaYF_4:Yb^{3+}/Er^{3+}$ UCNP synthesized at 190 °C as a function of different hydrothermal reaction times, (a) 2 h, (b) 3 h, (c) 5 h, (d) 7 h, (e) 8 h, (f) 10 h, (g) 15 h, and (h) 24 h.

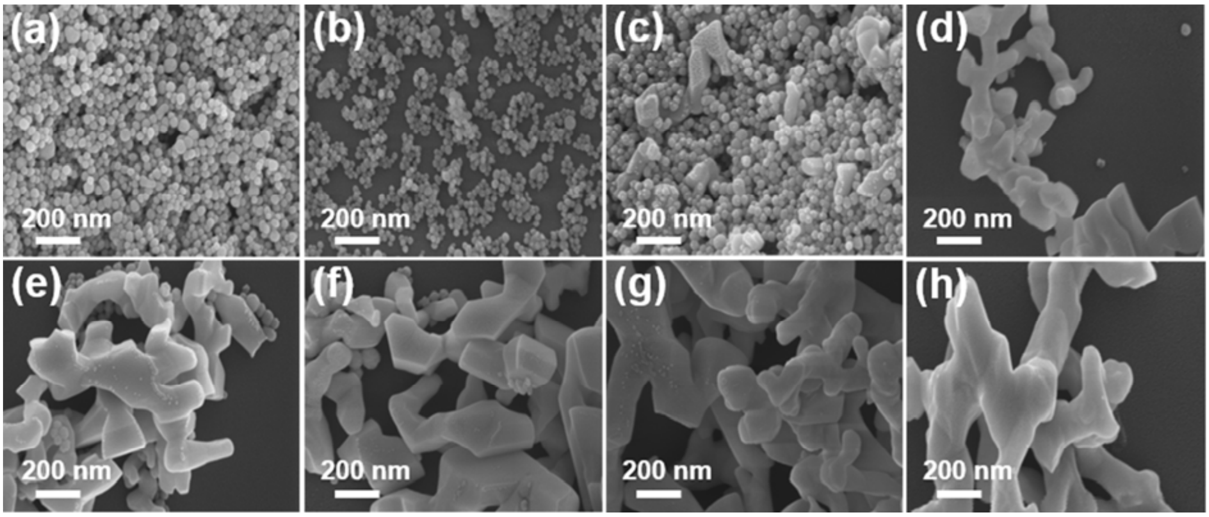

Fig. S3. SEM images of $NaYF_4:Yb^{3+}/Er^{3+}$ UCNP synthesized at 200 °C as a function of different hydrothermal reaction times, (a) 2 h, (b) 3 h, (c) 5 h, (d) 7 h, (e) 8 h, (f) 10 h, (g) 15 h, and (h) 24 h.

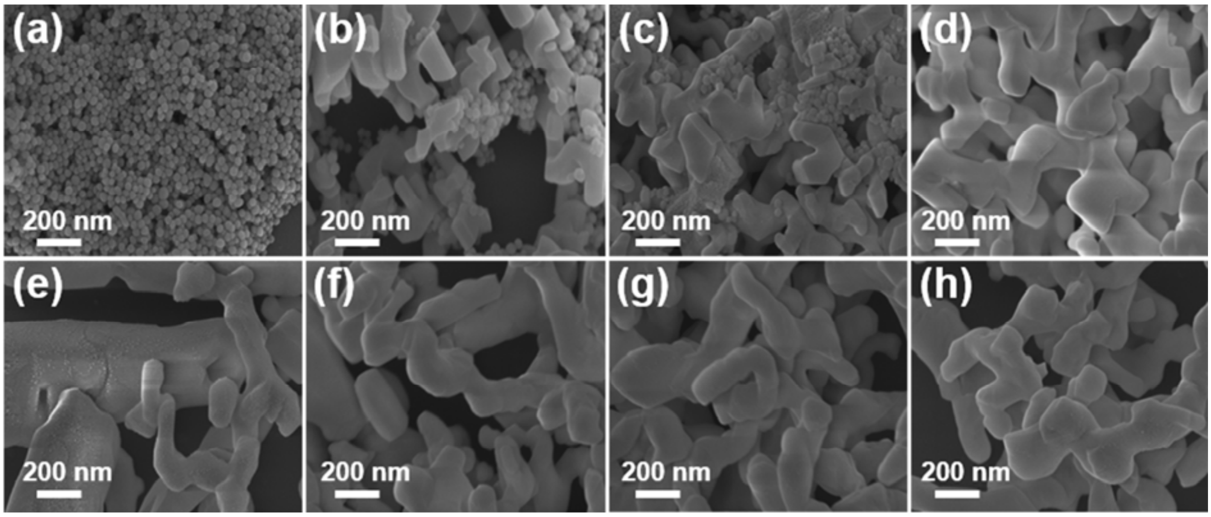

Fig. S4. SEM images of $NaYF_4:Yb^{3+}/Er^{3+}$ UCNP synthesized at 210 °C as a function of different hydrothermal reaction times, (a) 2 h, (b) 3 h, (c) 5 h, (d) 7 h, (e) 8 h, (f) 10 h, (g) 15 h, and (h) 24 h.

Particle size distribution of UCNPs at different hydrothermal reaction times and temperatures

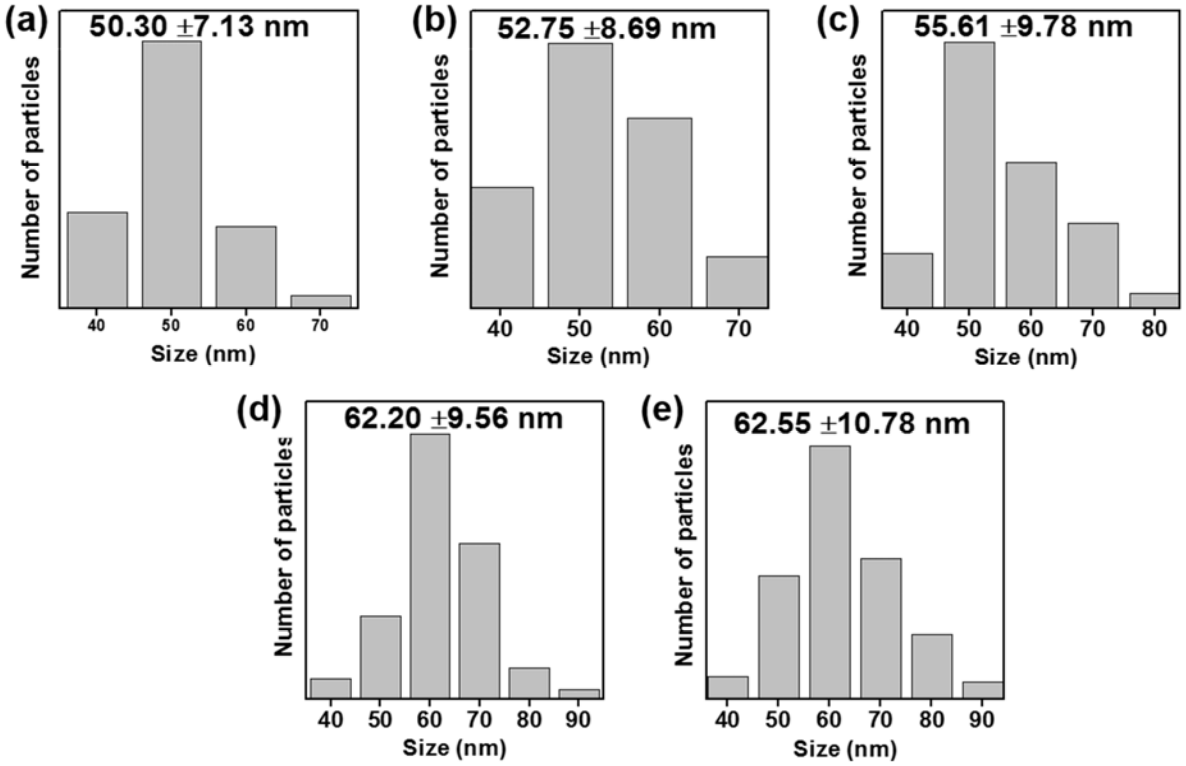

Fig. S5. Particle size distribution of $NaYF_4:Yb^{3+}/Er^{3+}$ UCNP synthesized at 180 °C as a function of different hydrothermal reaction times, (a) 2 h, (b) 3 h, (c) 5 h, (d) 7 h, and (e) 8 h.

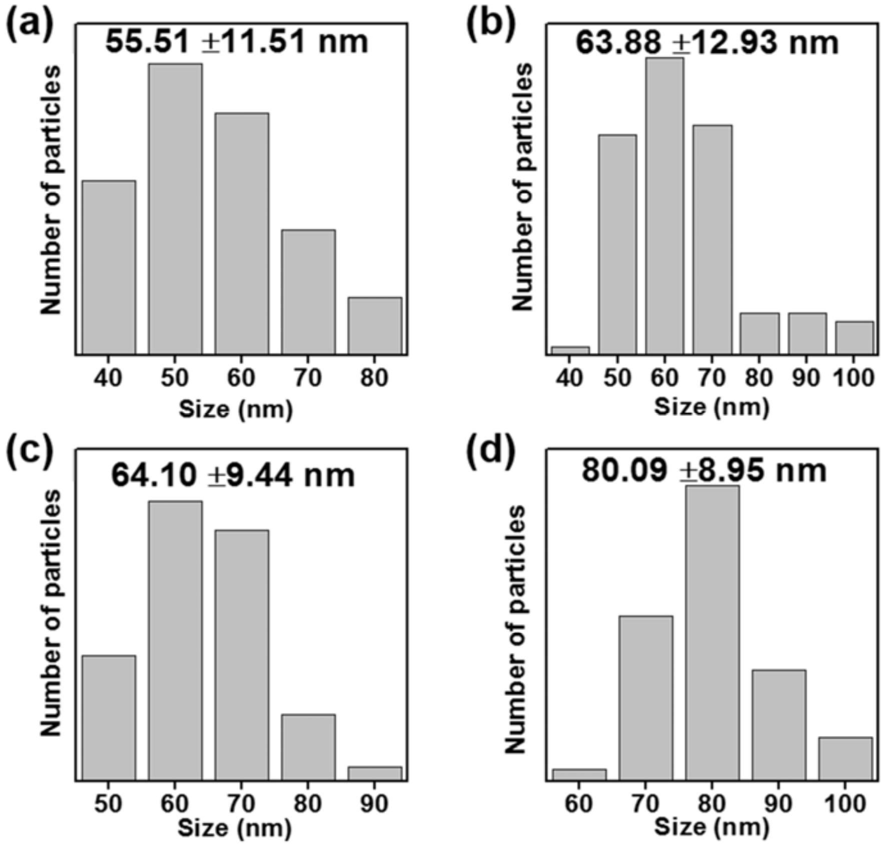

Fig. S6. Particle size distribution of $NaYF_4:Yb^{3+}/Er^{3+}$ UCNP synthesized at 190 °C as a function of different hydrothermal reaction times, (a) 2 h, (b) 3 h, (c) 5 h, and (d) 7 h.

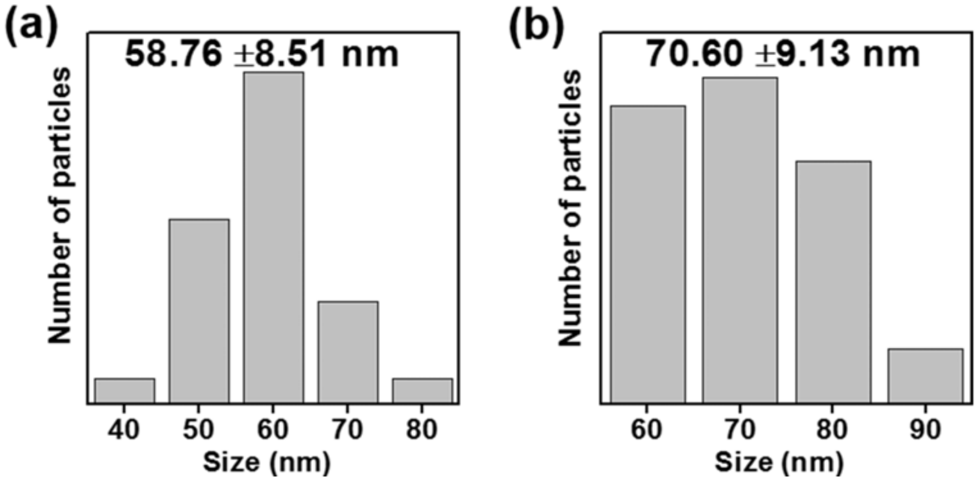

Fig. S7. Particle size distribution of $NaYF_4:Yb^{3+}/Er^{3+}$ UCNP synthesized at 200 °C as a function of different hydrothermal reaction times, (a) 2 h and (b) 3 h.

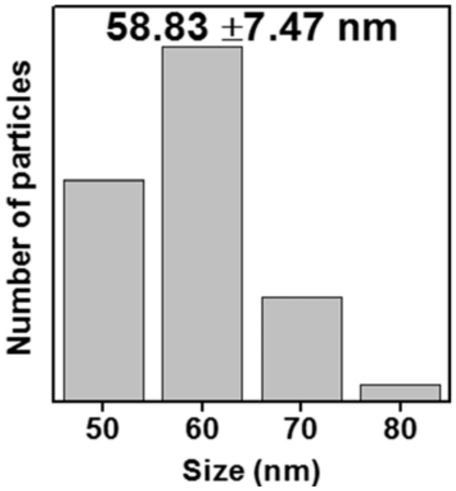

Fig. S8. Particle size distribution of $NaYF_4$:Yb^{3+}/Er^{3+} UCNP synthesized at 210 °C as a function of hydrothermal reaction time of 2 h.

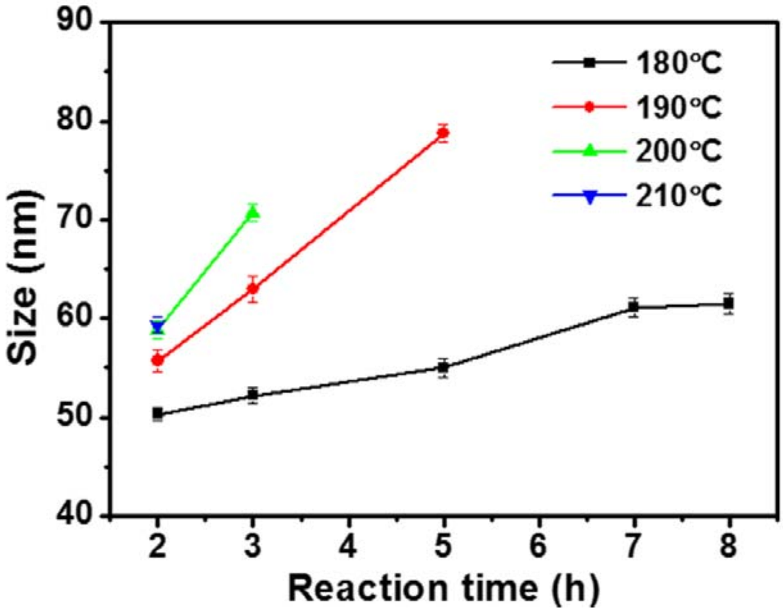

Fig. S9. Comparison of the particle size distribution of $NaYF_4$:Yb^{3+}/Er^{3+} UCNP synthesized at different hydrothermal reaction temperatures and times.

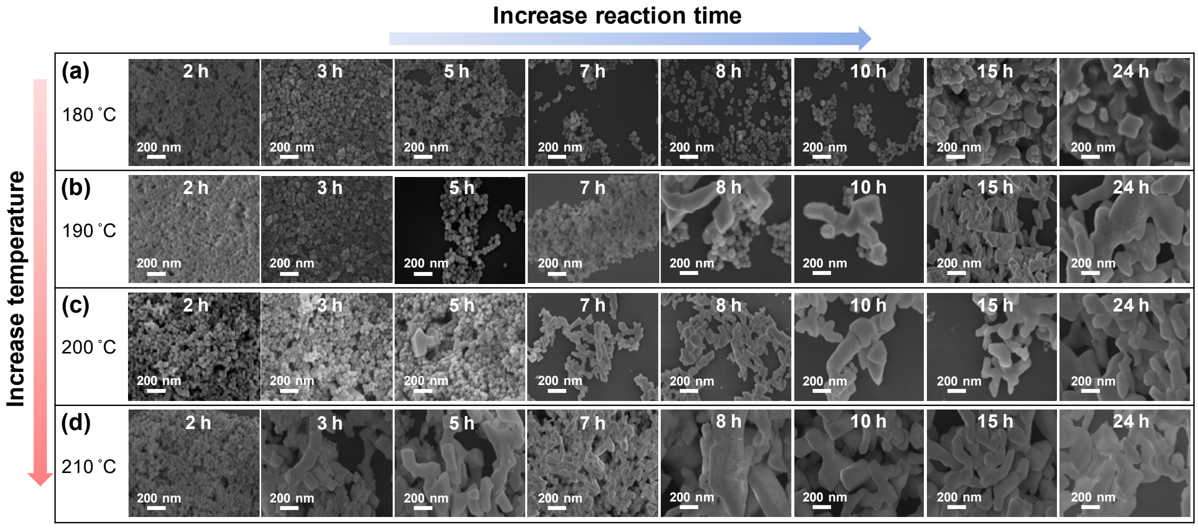

Fig. S10. SEM images show the reproducibility of the particles at different HR times and temperatures.

XRD patterns of UCNP at different hydrothermal reaction times and temperatures

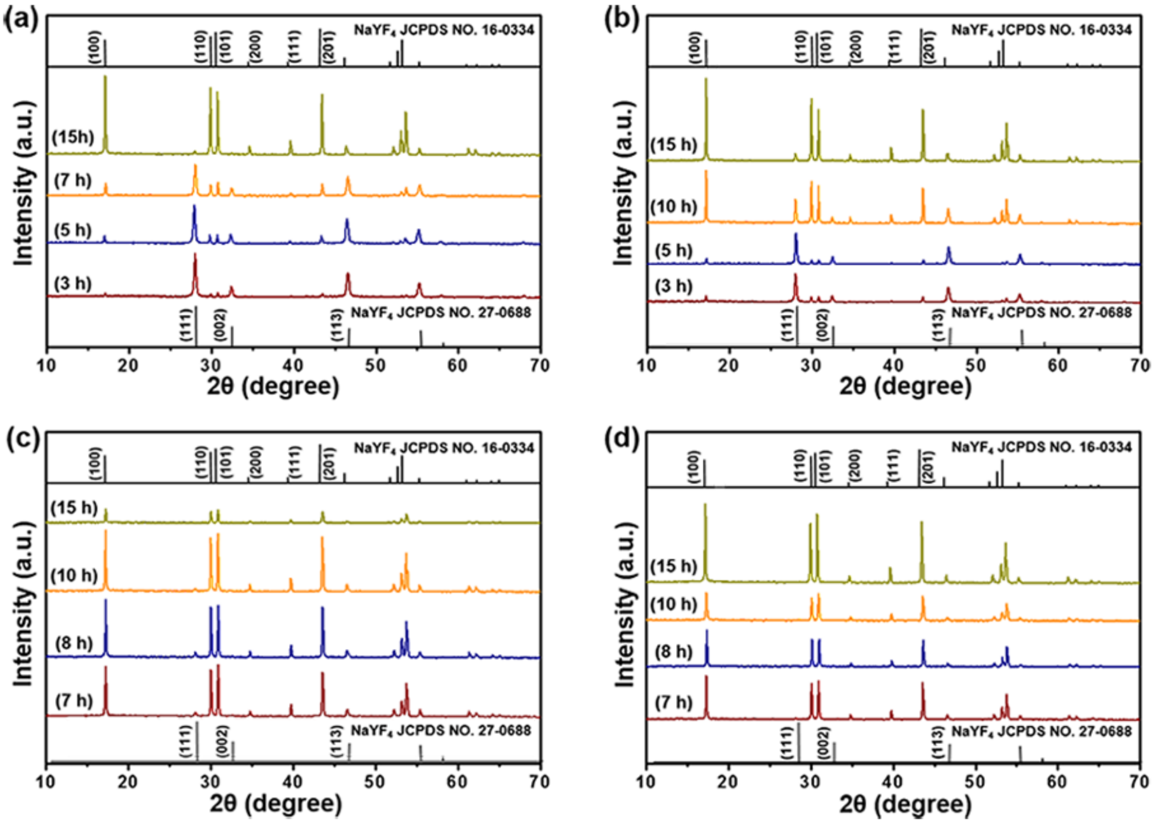

Fig. S11. XRD patterns of the synthesized NaYF4:Yb^{3+}/Er^{3+} UCNP (a) 180 °C, (b) 190 °C, (c) 200 °C, and (d) 210 °C. The particles corresponding to both α-NaYF4, PDF 00-027-0688 and β-NaYF4, PDF 00-016-0334.

STEM images of UCNPs at different hydrothermal reaction times and temperatures

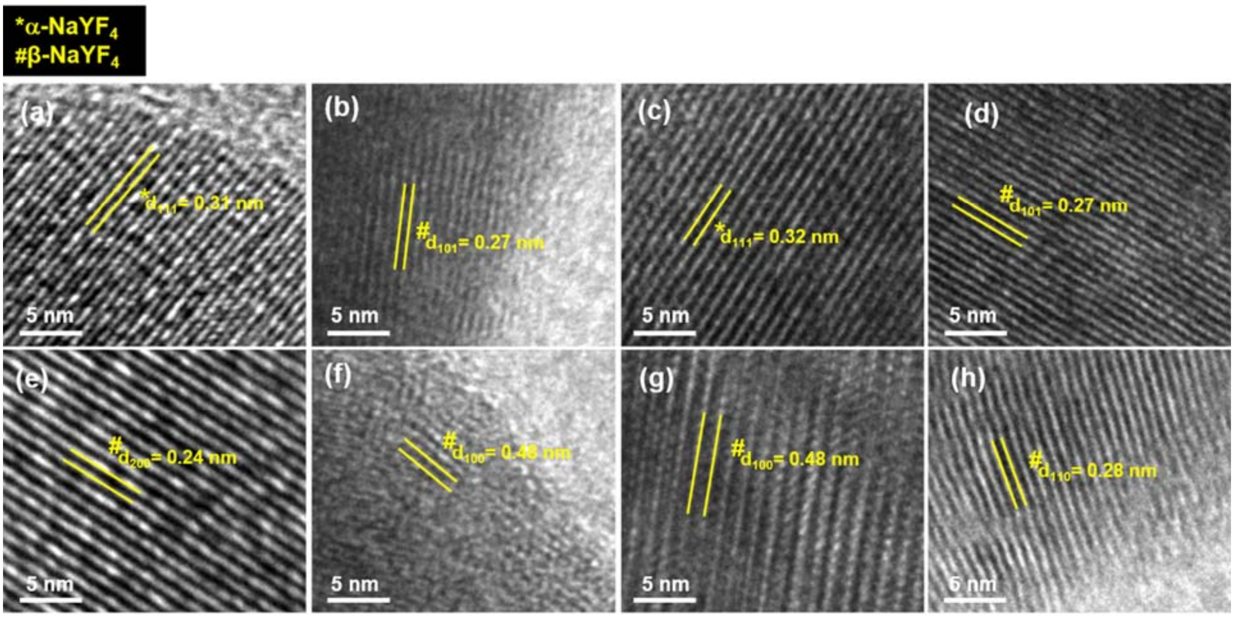

Fig. S12. STEM images of $NaYF_4$:Yb^{3+}/Er^{3+} UCNP synthesized at 180 °C for (a) 5 h and (b) 24 h, 190 °C for (c) 5 h and (d) 24 h, 200 °C for (e) 5 h and (f) 24 h, 210 °C for (g) 5 h and (h) 24 h.

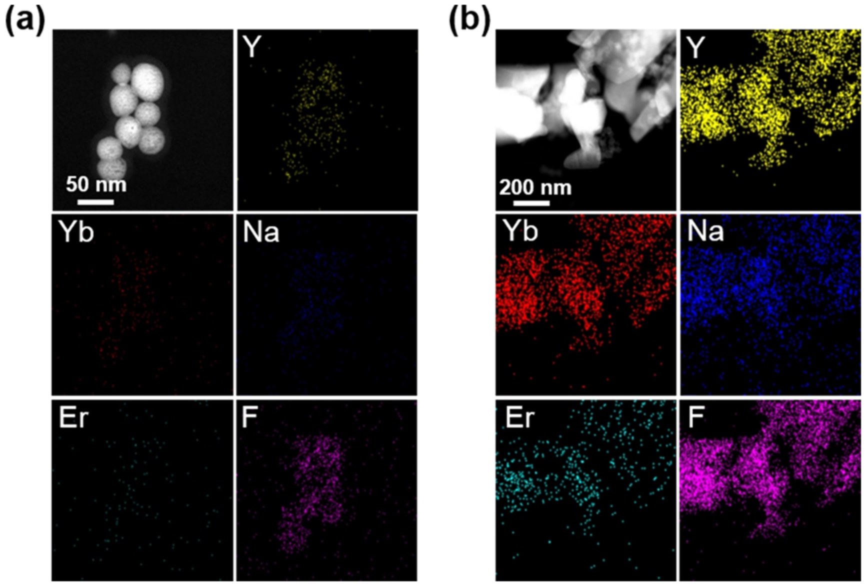

Fig. S13. High angle annular dark field-STEM images of $NaYF_4$:Yb^{3+}/Er^{3+} UCNP synthesized at 180 °C for (a) 5 h and (b) 24 h. Mapping of elements in synthesized UCNP; Y, Yb, Na, Er, F.

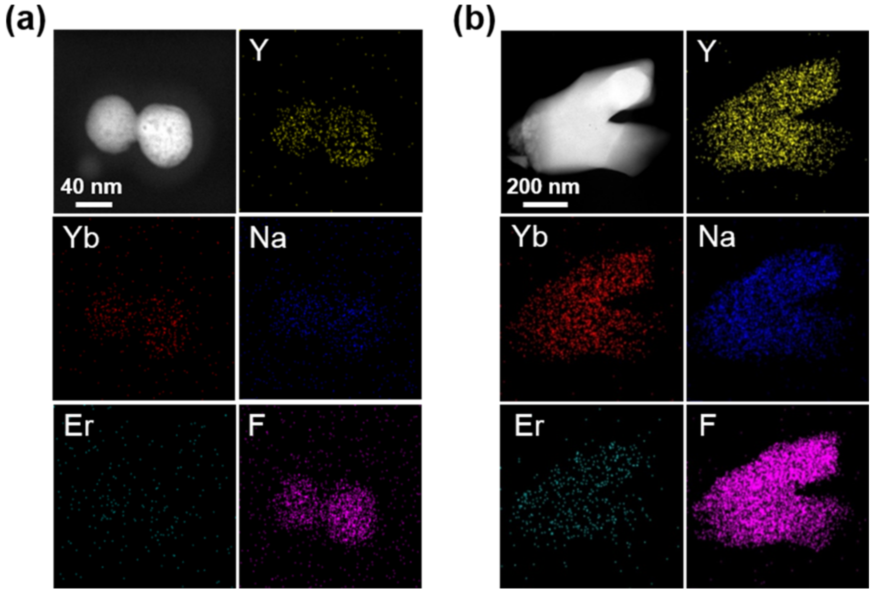

Fig. S14. High angle annular dark field-STEM images of $NaYF_4$:Yb^{3+}/Er^{3+} UCNP synthesized at 190 °C for (a) 5 h and (b) 24 h. Mapping of elements in synthesized UCNP; Y, Yb, Na, Er, F.

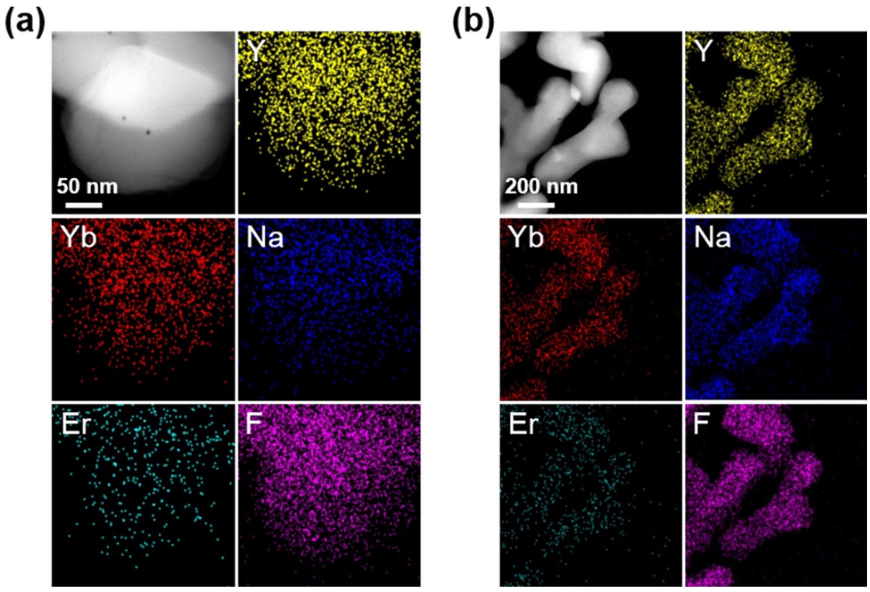

Fig. S15. High angle annular dark field-STEM images of $NaYF_4$:Yb^{3+}/Er^{3+} UCNP synthesized at 200 °C for (a) 5 h and (b) 24 h. Mapping of elements in synthesized UCNP; Y, Yb, Na, Er, F.

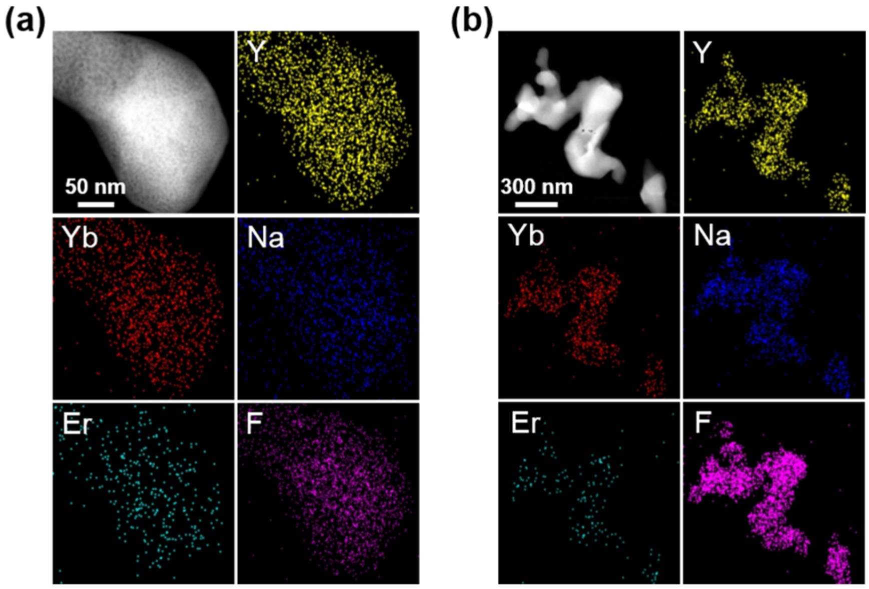

Fig. S16. High angle annular dark field-STEM images of $NaYF_4$:Yb^{3+}/Er^{3+} UCNP synthesized at 210 °C for (a) 5 h and (b) 24 h. Mapping of elements in synthesized UCNP; Y, Yb, Na, Er, F.

UC luminescence spectra of UCNPs at different hydrothermal reaction times and temperatures

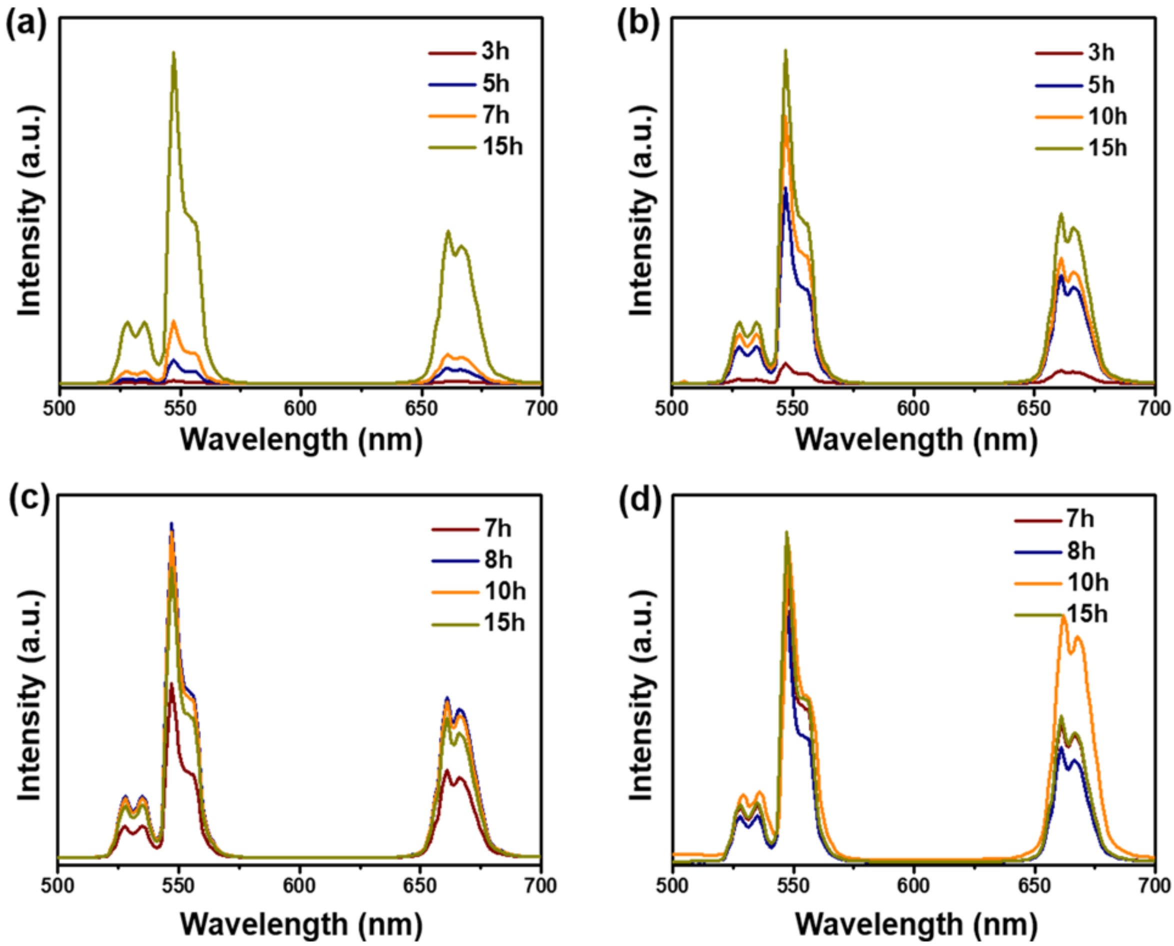

Fig. S17. NIR to visible UC luminescence spectra of $NaYF_4:Yb^{3+}/Er^{3+}$ UCNP synthesized at different hydrothermal reaction temperatures and times. Maximum peaks corresponding to green (527, 540 nm) and red (655 nm) emission under 980-nm laser diode excitation.

Time-resolved emission spectra of UCNPs at different hydrothermal reaction times and temperatures

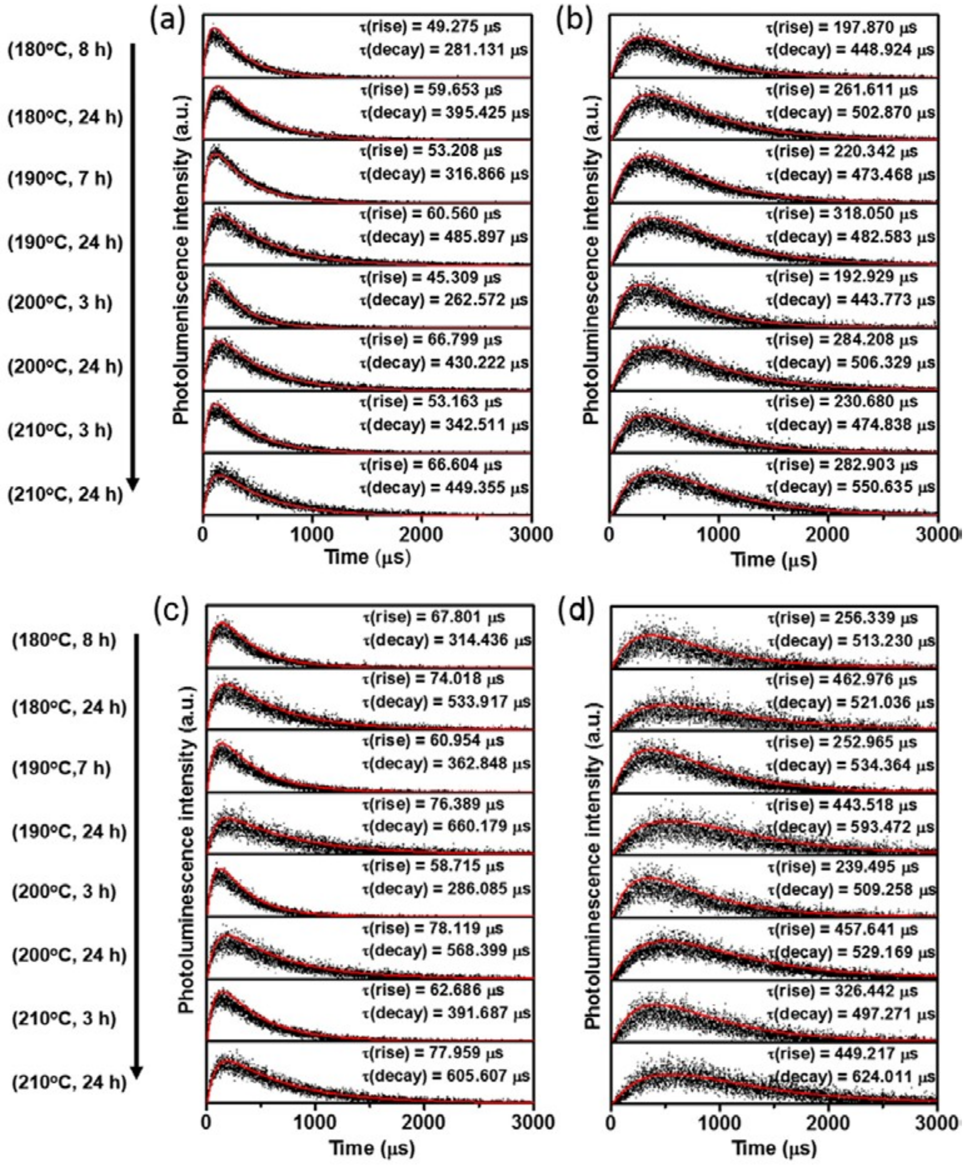

Fig. S18. Photoluminescence emission spectra of UCNPs (a, b) solution and (c, d) powder samples observed at (a, c) 540 nm for green emission bands, and (b, d) 655 nm for red emission bands under 980-nm laser excitation with different HR times and temperatures. The red solid lines highlighted the best fits to the time-resolved spectra.

Characterization of UCNP and UCNP@PAA

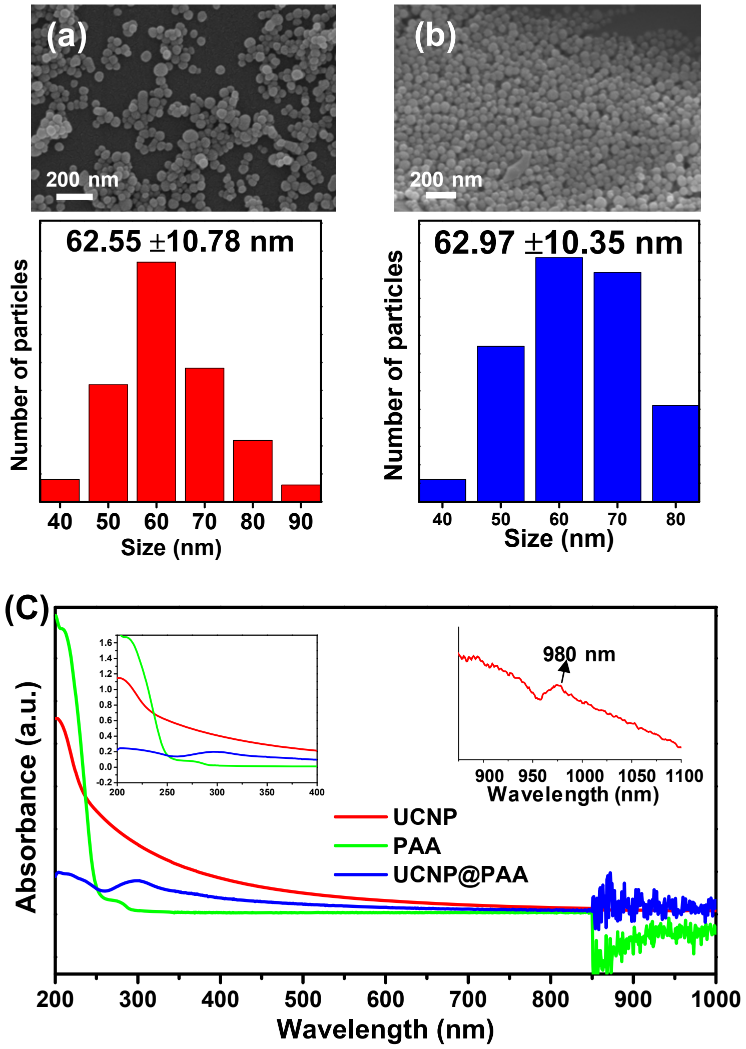

Fig. S19. SEM images of (a) UCNP (b) UCNP@PAA and (c) UV/Visible absorbance spectra of UCNP, PAA[1,2], and UCNP@PAA; insets show the more detailed UV/Vis spectra of UCNP, PAA, UCNP@PAA (left) and bared UCNP (right).

Dispersibility test of UCNP and PAA-UCNP in different solvents

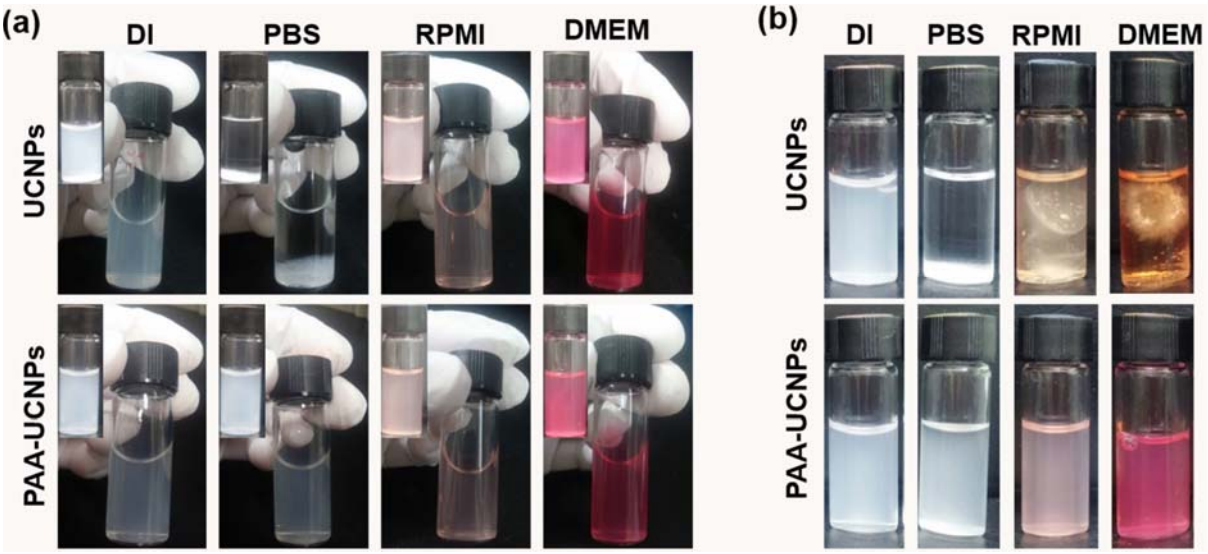

Fig. S20. Long-term stability of as-prepared UCNP and UCNP@PAA in different solvents after (a) one day and (b) one month after 5-s sonication.

***In vitro* cytotoxicity analysis of UCNP@PAA with different concentrations**

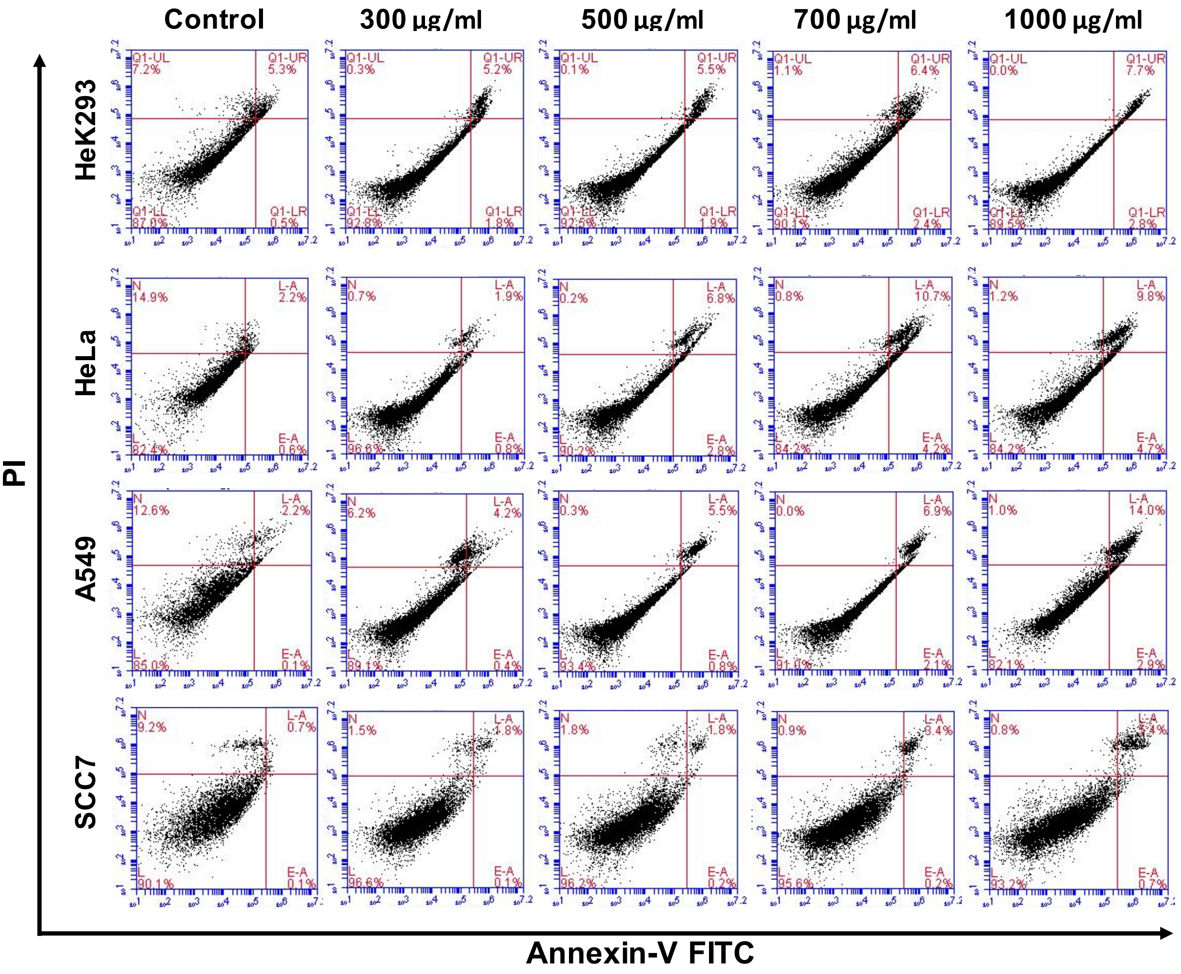

Fig. S21. Cell apoptosis and necrosis analysis of different cancer cell lines using flow cytometer after treatment with different concentration of UCNP@PAA. The different letters embedded in spectra described as L; live, N; necrosis, L-A; late apoptosis, E-A; early apoptosis.

Fluorescence image of HeLa cells

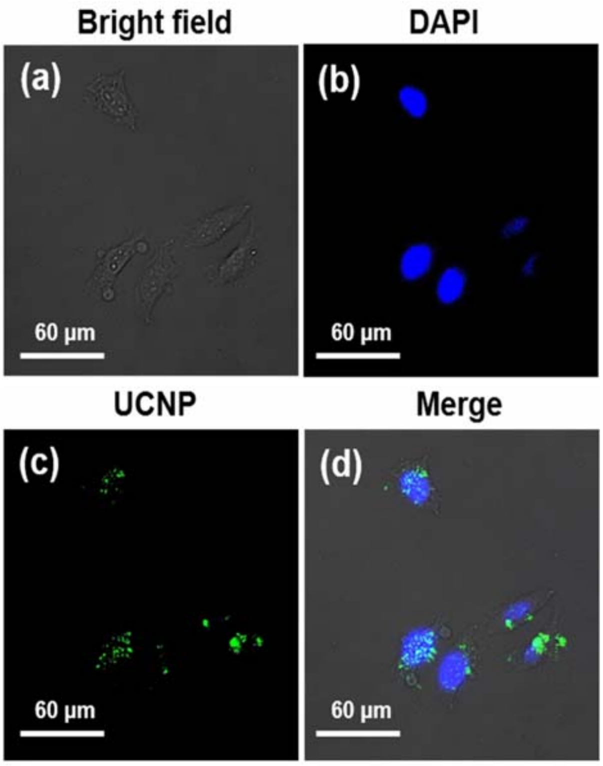

Fig. S22. Fluorescence images of HeLa cells treated with 100 µg/ml of UCNP@PAA and incubated for 4 h: (a) bright field, (b) DAPI staining, (c) UC luminescence, (d) merged.

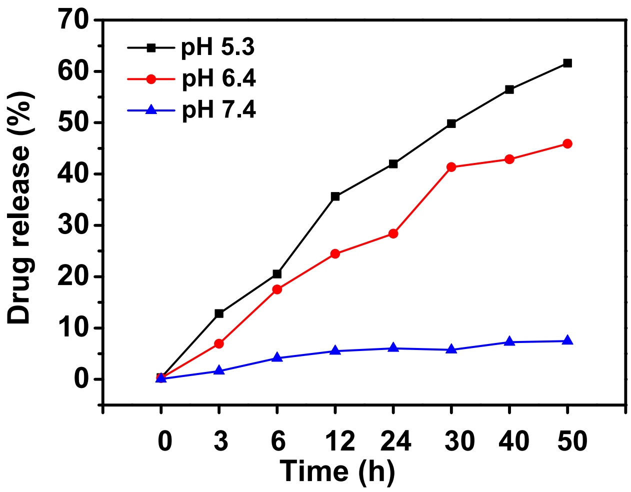

Fig. S23. Drug releasing efficacy of UCNP@PAA-DOX at different pHs and incubation times.

Dose response analysis

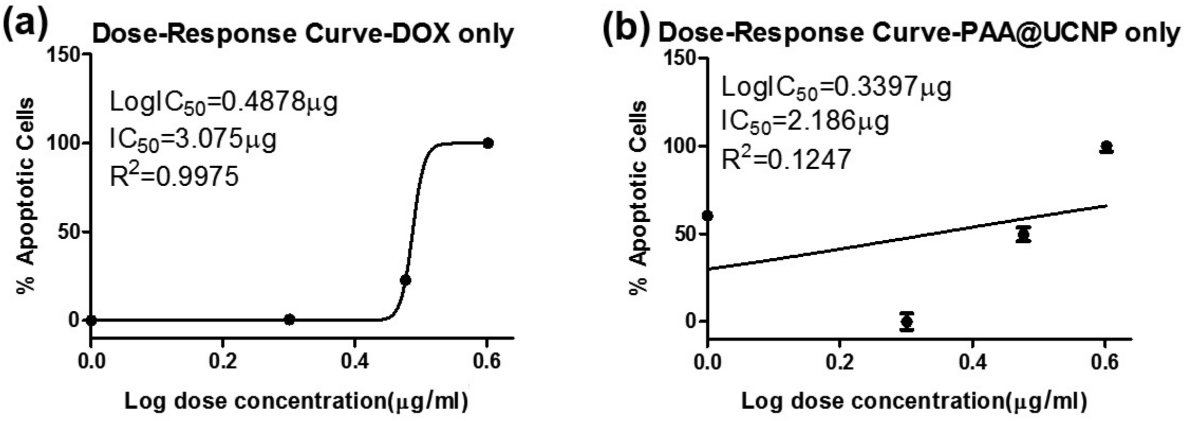

Fig. S24. IC_{50} of different cell lines in the presence of (a) DOX and (b) UCNP@PAA.

Supplementary references

1. Todica, M., Stefan, T., Simon, S., Balasz, I., Daraban, L. UV-Vis and XRD investigation of graphite-doped poly (acrylic) acid membranes. *Turk. J. Phys.* **38**, 261-267 (2014).
2. Gonzaga, V., Chrisostomo, B., Poli, A., Schmitt, C. Preparation, characterization and photostability of nanocomposite films based on poly (acrylic acid) and montmorillonite. *Mat. Res.* **21**, e20171024 (2018).
